# G protein stoichiometry dictates biased agonism through distinct receptor-G protein partitioning

**DOI:** 10.1038/s41598-017-07392-5

**Published:** 2017-08-11

**Authors:** Lauriane Onfroy, Ségolène Galandrin, Stéphanie M. Pontier, Marie-Hélène Seguelas, Du N’Guyen, Jean-Michel Sénard, Céline Galés

**Affiliations:** 1 Institut des Maladies Métaboliques et Cardiovasculaires, Institut National de la Santé et de la Recherche Médicale, U1048, Université de Toulouse, F-31432 Toulouse, France; 2 Independent Researcher Ottawa, Ottawa, ON Canada; 3 Service de Pharmacologie Clinique, Centre Hospitalier Universitaire de Toulouse, Faculté de Médecine, Université de Toulouse, F-31000 Toulouse, France

## Abstract

Biased agonism at G protein coupled receptors emerges as an opportunity for development of drugs with enhanced benefit/risk balance making biased ligand identification a priority. However, ligand biased signature, classically inferred from ligand activity across multiple pathways, displays high variability in recombinant systems. Functional assays usually necessity receptor/effector overexpression that should be controlled among assays to allow comparison but this calibration currently fails. Herein, we demonstrate that Gα expression level dictates the biased profiling of agonists and, to a lesser extent of β-blockers, in a Gα isoform- and receptor-specific way, depending on specific G protein activity in different membrane territories. These results have major therapeutic implications since they suggest that the ligand bias phenotype is not necessarily maintained in pathological cell background characterized by fluctuations in G protein expression. Thus, we recommend implementation of G protein stoichiometry as a new parameter in biased ligand screening programs.

## Introduction

G protein-coupled receptors (GPCRs) represent primary targets for drug development. However, many of these drugs display adverse side effects that can temper their clinical use. In recent years the concept of biased agonism has emerged and offers the possibility of alleviating and having more accurate control of this problem. Indeed, this now well-accepted paradigm defines the capability of a ligand to stabilize different conformations of a receptor and thus to activate or block a specific subset of intracellular associated signaling pathways.

This multitude of ligand-specific effects from a single receptor, also known as ligand texture, paves the way for the development of pathway-selective drugs with increased efficacy and less adverse effects^1^. This concept has been recently taken into consideration by pharmaceutical companies with several clinical trials based on the use of G protein- or β-arrestin-biased ligands currently in the pipeline^2^. Despite biased-agonism is more often contemplated from a clinical benefit standpoint, it could however as well contribute to an adverse benefit/risk balance in response to some drugs. Thus, β-blockers, originally developed to uniformly antagonize β-adrenergic receptors and currently considered as first-line drug in heart failure, have only recently been identified as biased ligands^3^ which could explain, in part, disparities in clinical efficacy among β-blockers^4–6^. Interestingly, the pharmacological imprinting of a biased ligand seems to be not only a fixed attribute of the chemical molecule but depends also on the cellular conditions. For instance, one study reported that classical antipsychotic drugs commonly antagonize the D2-R/β-arrestin pathway^7^ which could refer to their efficacy, while, conversely, another demonstrated partial agonism in a similar cell system^8^. Likewise, the angiotensin II-mimicking peptide SII was originally described as a β-arrestin-biased AT1-R agonist with no activity on G protein signaling^9^ but afterwards was shown to exhibit partial agonist efficacy on the G protein as well^10^. Thus, biased agonism remains a fickle and still poorly comprehended phenomenon, the underlying molecular basis of which needs further exploration.

Until now, the pharmacological definition of biased agonists has been assigned to their effects on stabilizing of different receptor conformations^11^. Recently, temporal control of the different signaling pathways also appeared as an important determinant in differently modulating the biased-profiling of a ligand^12, 13^. From a molecular standpoint, biased agonism redefines the pharmacological classification of ligand efficacy such that it no longer relies on a ligand/receptor bipartite but a more intricate ligand/receptor/effector tripartite, highlighting the importance of the nature of the effector. In classical GPCR pharmacology, the influence of the relative receptor-G protein effector stoichiometry on ligand efficacy has been previously underlined^14–18^, however, this notion has surprisingly never been evaluated in terms of delineating the biased activity of ligands. On the contrary, evaluation of ligand biased activity in recombinant systems generally relies on the comparison of different signaling pathways, each of which are probed with different levels of receptor or effector without calibration (*i.e*. II messenger production in cells stably expressing a receptor *versus* β-arrestin recruitment to a transiently expressed receptor) which should preclude any comparison and biased activity quantification^19^. This could in part account for the discrepancies in biased activity described for some ligands. Moreover, abnormal stoichiometry of the receptor-effector system has long been viewed as a specificity of recombinant cell systems allowing higher expression levels compared to natural ones^14^. However, quantification of receptors or effectors in natural systems has mostly relied on studies using tissues that express different cell types, thus preventing accurate and specific quantification for each cell type. In addition, physiological receptor-effector stoichiometry is not static, for instance, Gpr176 is expressed in a circadian manner by suprachiasmatic nucleus neurons^20^. Finally, both receptor and effector expression also undergo modifications in pathological contexts. Thus, G protein^21^ as well as GPCR expression profiles^22^ are profoundly modified during immune cell maturation. Some studies have also already described the downregulation of receptors, G proteins and secondary effectors in heart failure^23–25^, while other alterations in G protein expression have been reported in cancer^26^ and Parkinson’s disease^27^. This pathological cell environment should be better considered from a clinical biased-drug perspective and has rarely been systematically considered for *in vitro* drug screening.

In this study, we took advantage of HEK293T recombinant cell system that allows high flexibility in protein expression to perform the first systematic calibrated study evaluating and accurately quantifying the effect of varying the expression levels different heterotrimeric G proteins, the common effectors of all GPCR families, on the efficacy of GPCR ligands on G protein activation. We show that the Gα subunit expression levels plays a prominent role in the biased profiling of β-agonists but also antagonists, affecting both their potency and/or efficacy by targeting different membrane distribution of receptor-G protein populations. More surprisingly, we found that, in the resting state, the level of Gα expression influences the partitioning of not only Gα but also the co-expressed receptor in different cholesterol-enriched membrane domains. Thus, our data provide critical insights toward quantification of biased agonism but also improve our knowledge on the G protein/receptor interplay at resting state.

## Results

### Alterations of Gα subunits gene expression in failing cardiomyocytes

**Figure 1:**
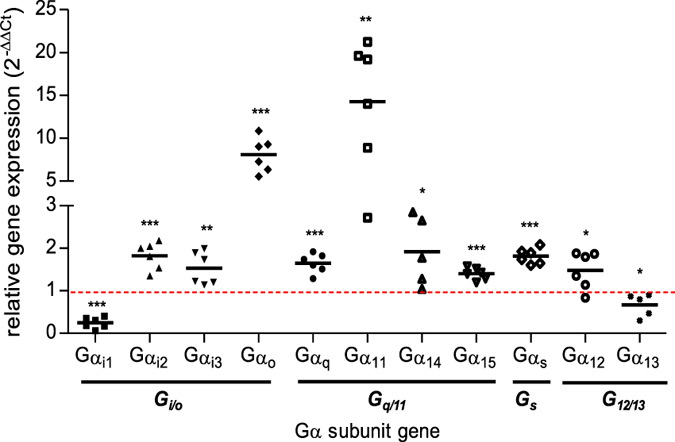
Quantification of heterotrimeric G protein α subunits gene expression levels in hypertrophic cardiomyocytes. Comparative analysis of Gα subunit gene expression of the four G protein families (G_i/o_, G_q/11_, G_s_, G_12/13_) in total RNA extracts from cardiomyocytes isolated from mice exhibiting or not (control) transversal aortic banding (TAC) obtained by Fluidigm Real-time qPCR. Results are expressed as the relative induction of the genes in C57Bl/6 mice submitted to TAC for 15 days (n = 6) compared to control mice (n = 6) wherein genes expression are equal to 1. The statistical significance of the change in expression level was assessed using unpaired Student’s t-test. *P < 0.05, **P < 0.01, ***P < 0.001.

We first wondered whether Gα subunit expression, the GTPase core of heterotrimeric G proteins, was prone to modifications in specific cells from pathological tissue. We thus evaluated the relative expression of the major Gα subunits from all G protein families (*Gi/o*: αi1, αi2, αi3, αo - *Gs:* αsL - *Gq/11:* αq, α11, α14, α15 - *G12/13:* α12, α13) in cardiomyocytes isolated from mice with heart failure secondary to an experimental barometric stress induced by transverse aortic constriction previously described^28^. Quantitative PCR led to the identification of important modifications in Gα gene expression as shown in Fig. 1. Specifically, mRNA encoding Gαo and Gα11 were 7.5 and 12.5 fold over-expressed respectively in failing cardiomyocytes compared to control cardiomyocytes. All other Gα subunit mRNAs were also prone to a significant but less substantial up-regulation oscillating between 1.5 and 2 fold, with the exception of the Gαi1 but also Gα13 subunits which were significantly down-regulated. These results clearly indicate that the gene expression of the Gα subunits is highly prone to important variation in diseased cardiomyocytes, most likely suggesting correlated modifications in the resultant Gα proteins. Based on these results, we then decided to explore the relationship between ligand pharmacology and G protein stoichiometry more specifically by focusing on Gαi1, Gαo, Gα11, and Gαs as representative Gα isoforms modulating their expression to different degrees.

### Gα stoichiometry dictates the maximal efficacies of different ligands

**Figure 2:**
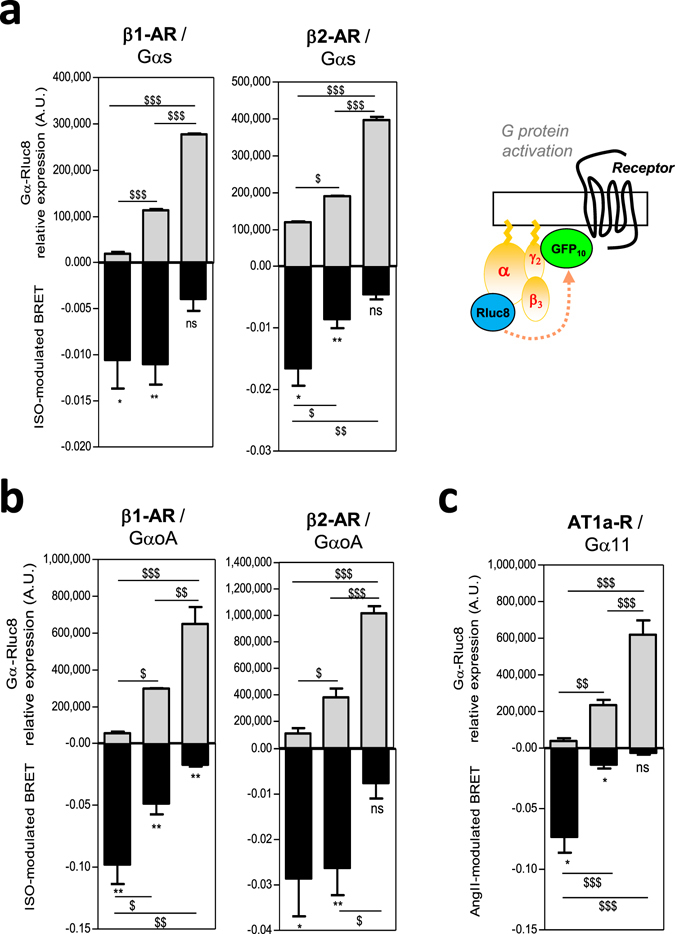
Gα subunit expression level impacts on agonist-mediated G protein activation. (**a,b**) BRET (black) in HEK293T cells co-expressing HA-β1-AR (left panels) or HA-β2-AR (right panels) or (**c**) AT1a-R receptors and different expression levels of Gαs-Rluc8 (**a**) or GαoA-Rluc8 (**b**) or Gα11-Rluc8 (**c**) in the presence of fixed GFP10-Gγ2 and Gβ3 untagged subunits. Cells were stimulated or not for 1 min with 10 µM of the indicated selective agonists isoproterenol (ISO) or angiotensin II (AngII). Results are expressed as the difference in BRET signals measured in presence and absence of ligand. Data represent the mean ± s.e.m. of at least three independent experiments. The statistical significance between stimulated and unstimulated cells was assessed using paired Student’s t-test (*P < 0.05, **P < 0.01, ***P < 0.001). Gα-Rluc8 relative expression levels (gray) were depicted by the luminescence measurement and represent the mean ± s.e.m. of at least three independent experiments. The statistical significance of the difference in luminescence between Gα expression levels was assessed using one-way ANOVA followed by a Tukey’s multiple comparison test (^$^ *P* < 0.05, ^$$^ *P* < 0.01, ^$$$^ *P* < 0.001).

We first performed a preliminary screen in recombinant HEK293T cells to test the consequences of variable Gα subunits expression on their activation. Gαs and GαoA activation was measured following synthetic isoproterenol (ISO)-stimulation of the β-adrenergic (β1-AR or β2-AR) receptors while Gα11 activation was measured following angiotensin II (AngII)- stimulation of the angiotensin II (AngII) type 1 receptor (AT1-R). These GPCRs are all prominent drug targets in heart failure. Direct activation of the specific Gα subunits was assessed using BRET^2^ probes which we previously described^10, 29^ by measuring the interaction between the specific Gα-Rluc8 and GFP10-Gγ2 subunits in the presence of the complementary untagged Gβ3 which is specifically expressed in adult cardiomyocytes^30^. Indeed, these G protein activity-BRET probes provide powerful and accurate tools to delineate ligand pharmacology. Thus, we have previously shown that they can not only allow sense the agonist efficacy of ligands, reflected by a decrease in the BRET signal^10, 29, 31^ (negative BRET modulation), but conversely can also detect inverse agonist efficacy, measured as an increase in the BRET signal^32^ compared to the basal BRET signal that is indicative of preassembled inactive Gαβγ complexes. Thus, G protein activation was evaluated under different Gα-Rluc8 expression levels that were systematically quantified by measuring the total amount of luminescence. In these experiments, we used receptor excess and saturating ligand concentration to ensure depicting of the maximal ligand/receptor/G protein complexed fractions of the receptor population. Reference agonist ligands (*i.e*. ISO or AngII) were applied for 1 min in order to stimulate the β1-AR or β2-AR/Gαs (Fig. 2a), β1-AR or β2-AR/GαoA (Fig. 2b) or AT1-R/Gα11 (Fig. 2c) complexes. Unexpectedly, in all of the conditions tested, higher levels of Gα subunit expression correlated with reduced ligand-induced negative BRET modulation (Fig. 2). These results are particularly unexpected in a model that classically predicts an increased in receptor-G protein coupling when G proteins are in excess. Nevertheless, they support the idea that high G protein expression levels could affect the pharmacological profile and the efficacy of a given ligand.

### Gα isoform and stoichiometry dictate varying β-adrenergic ligand pharmacology

To gain further insight into the influence of Gα expression level on ligand efficacy, we decided to focus on the β1- and β2-AR receptors and Gαs, Gαi1 and GαoA activation promoted by the reference synthetic agonist ISO, as well as two natural agonists of these GPCRs, epinephrine (EPI) and norepinephrine (NE).

We first measured the activation of these different G protein activity-BRET biosensors in HEK293T cells in the absence of β1- and β2-AR over-expression to evaluate the potential contribution of endogenous adrenergic responses (Supplementary Fig. 1). Importantly, G protein activation was evaluated with both high and low Gα expression levels that were tightly calibrated to similar levels in each transfection for all subunits (Supplementary Fig. 1, inset). It is also worth noting that, while the various Gα-Rluc8 subunits used in these experiments were over-expressed at two distinct expression levels, the overall expression of these recombinant proteins was still below the expression level of the corresponding endogenous Gα subunit, as shown by western-blotting in the case of the Gαs subunit (Supplementary Fig. 2). As expected, although we did not detect any significant Gαs activation, except in the presence of EPI at high Gαs expression levels (Supplementary Fig. 1a), EPI promoted a significant activation of both Gαi1 and GαoA whereas NE more selectively activated GαoA and ISO was ineffective (Supplementary Fig. 1b,c), thus confirming the presence of endogenous adrenergic receptors as known in HEK293T cells. However, the endogenous receptors were not problematic when both G protein biosensors and receptors were over-expressed, since under these experimental conditions, the G protein activation response was always shifted toward the over-expressed receptor (R)-G protein (G) complex. Indeed, when we sensed G protein activation in the presence or absence of β1-/β2-AR (Supplementary Fig. 3a) or conformational rearrangements within the different R-G complexes (Supplementary Fig. 3b) by measuring BRET between Gα-Rluc8 and β1-AR-GFP10 or β2-AR-GFP10 as previously described^10, 29^, we found a good correlation between maximal agonist responses in R-G conformational changes and G protein activation profiles obtained with over-expressed β1-AR or β2-AR (Supplementary Fig. 3c). In contrast, no parallel could be found between conformational changes stabilized by β-agonists on the Gαi1/β1- or β2-AR complexes and G protein activation profiles obtained with endogenous adrenergic receptors (Supplementary Fig. 3d). Moreover, the maximal Gαi1 activation response promoted by the different β-AR agonists in the presence of over-expressed β1- or β2-AR agreed with the rank order selectivity of ligand potency for these receptors with EPI > NE for β2-AR while NE > EPI for β1-AR. These results were also corroborated by cAMP measurements (Supplementary Fig. 4) performed in the presence or not of Gαs, β1- or β2-AR over-expression under conditions similar to those of the BRET experiments that measured the G protein activation. EPI and NE promoted significant concentration-dependent cAMP production with endogenous receptors but with a low potency that was always leftward shifted and was associated with marked increase in maximal efficacy when the β1- or β2-AR were over-expressed (Supplementary Fig. 4; Supplementary Table 1). Further addition of the Gαs subunit potentiated the maximal efficacy of EPI and NE independently of its expression level at β1-AR (Supplementary Fig. 4a) but not at β2-AR (Supplementary Fig. 4b), indicative of a highly selective influence of Gα on receptor-mediated adenylyl cyclase activation.

**Figure 3:**
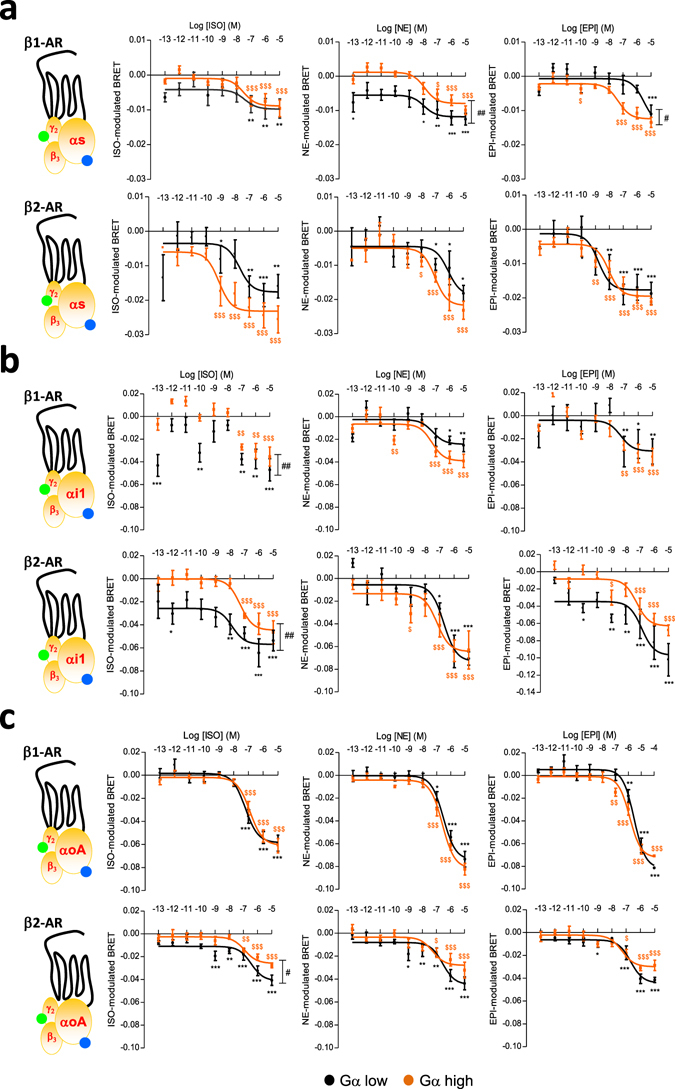
Influence of the Gα subunit expression level on the concentration-response curves of β-AR agonists-mediated G protein activation. (**a**–**c**) BRET in HEK293T cells co-expressing HA-β1-AR (upper panels) or HA-β2-AR (lower panels) receptors and low (black) or high (orange) expression levels of Gαs-Rluc8 (**a**), Gαi1-Rluc8 (**b**) or GαoA-Rluc8 (**c**) in presence of fixed GFP10-Gγ2 and Gβ3 untagged subunits. Cells were stimulated or not for 1 min with increasing concentrations of the indicated agonists (Isoproterenol, ISO; Norepinephrine, NE; Epinephrine, EPI). Results are expressed as the difference in BRET signals measured in presence and absence of ligand. Data represent the mean ± s.e.m. of at least four independent experiments. The statistical significance between unstimulated cells and cells stimulated with the different ligand concentrations in low (*) and high ($) Gα-Rluc8 conditions was assessed using one-way ANOVA followed by a Dunnett’s multiple comparison test (*P < 0.05, **P < 0.01, ***P < 0.001). The statistical significance between low and high dose-response curves was assessed using two-way ANOVA followed by a Bonferroni post-test (^#^ *P* < 0.05, ^##^ *P* < 0.01).

**Figure 4:**
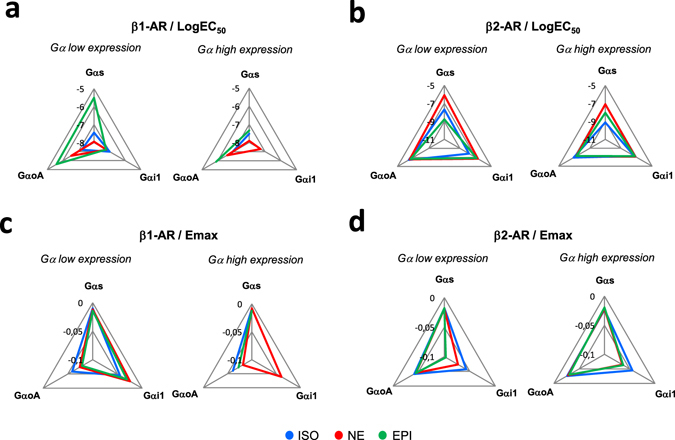
Schematic multi-axial profiling of β-AR agonist efficacies and potencies at G protein activation with different Gα expression levels. (**a**–**d**) ISO, NE or EPI potencies (**a**,**b**) or maximal efficacies (**c**,**d**) at G protein (Gαs, Gαi1, GαoA) activation upon stimulation of β1-AR (**a**,**c**) or β2-AR (**b**,**d**) presented in Fig. 3 and Supplementary Tables 2 and 3 are shown in a web.

We then examined the pharmacological activation profile of the three Gα subunits, Gαs, Gαi1 and GαoA, when expressed at high and low levels in the presence of β1- or β2-AR (Fig. 3). In these experiments, each receptor was calibrated to ensure similar cell surface expression levels for each of the three Gα subunits experimental condition (either high and low expression) but not between receptors (Supplementary Fig. 5), while all Gα subunits expression levels (high or low) were similarly calibrated between the three isoforms (Supplementary Fig. 6). For Gαs activation, both NE and EPI displayed different dose-response curves in the presence of β1-AR depending on Gα expression levels (Fig. 3a). When compared to low levels of Gαs expression, high levels specifically increased EPI potency, but decreased the maximal efficacy of NE (Supplementary Table 2). Interestingly, while the ISO response was insensitive to Gαs levels with β1-AR (Fig. 3a), high Gαs levels increased ISO-potency at β2-AR (Fig. 3a; Supplementary Table 3). For Gαi1 activation by β1-AR (Fig. 3b), ISO displayed an atypical pharmacological profile with a double-bell-like shaped dose-response curve showing significant activation at very low ligand concentrations in the presence of low Gαi1 levels. On the contrary, the expression level of Gαi1 had no significant impact on NE and EPI responses. In the presence of β2-AR (Fig. 3b), whereas Gαi1 activation was detected at low Gαi1 levels in the low ISO and EPI concentration ranges and specifically potentiated the maximal activation promoted by these two ligands, Gαi1 levels had no detectable influence on the NE response. Although GαoA belongs to the same Gi/o family as Gαi1, the dose-response curve for each ligand was quite different (Fig. 3b,c). ISO, NE and EPI displayed similar concentration-response profiles with β1-AR in the presence of either low or high GαoA levels while for β2-AR, low GαoA levels increased the maximal efficacy of all three ligands (Fig. 3c). Interestingly, when similar Gα stoichiometry experiments were conducted with some β-blockers used (bisoprolol and metoprolol) or not (timolol) in the treatment of patients with heart failure, results, despite more fluctuating, highlighted lesser susceptibility of this pharmacological class toward Gα level compared to β-agonists (Supplementary Fig. 7). When considering Gαs, no significant activation was measured for none of the three ligands through β1- or β2-AR, independently of the Gαs level, in agreement with their antagonistic properties despite some agonist efficacy could be depicted at high timolol concentrations for high Gαs expression. Agonist efficacy of β-blockers was more evident at Gαi1 and GαoA activation and more likely insensitive to the Gα expression level although low levels of some Gα can exacerbate the agonist dose-response but with high variability (BISO/β2-AR/Gαi1; BISO/β1-AR/GαoA; METO/β2-AR/GαoA). The LogEC50 and Emax values obtained with the β-agonists have been graphically represented in a web format to better appreciate the fingerprint of each β-adrenergic agonist in activating the three Gα subunits at both high and low expression levels for β1-AR and β2-AR (Fig. 4). All of the webs clearly highlight that low versus high Gα expression levels modified the bias fingerprint for almost all ligands and the two receptors, thus demonstrating that the expression level of the Gα subunit accurately dictates bias of ligands. It is worth noting that all three β-adrenergic agonists displayed different fingerprints from each other for each web, most likely indicating that they behaved as biased ligands.

### Gα isoform and stoichiometry dictates selective β-adrenergic agonist responses through different membrane R-G populations

**Figure 5:**
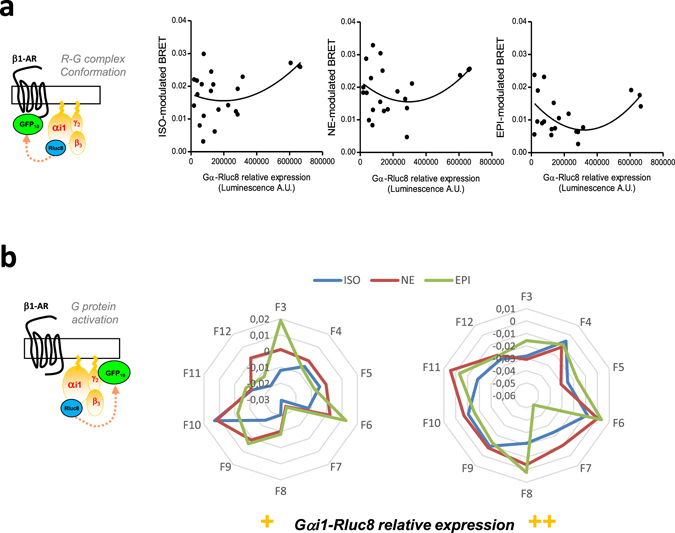
Influence of the Gα stoichiometry on β-adrenergic agonist responses through different membrane Receptor-G protein populations. (**a**) Receptor-G protein complexes conformations assessed by BRET in HEK293T cells co-expressing β1AR-GFP10 receptor and different expression levels of Gαi1-Rluc8 in presence of fixed Gγ2 and Gβ3 untagged subunits. Cells were then stimulated or not for 1 min with 10 µM of the indicated ligands (ISO, NE or EPI). Results are expressed as the difference in BRET signals measured in presence and absence of ligand and are representative of four independent experiments. Curves were generated using a polynomial quadratic model. (**b**) G protein activation measured in sucrose fractions by BRET in HEK293T cells, co-expressing HA-β1AR receptor and different expression levels of Gαi1-Rluc8 in presence of fixed GFP10-Gγ2 and Gβ3 untagged subunits, and processed for raft purification. Results are expressed as the difference in BRET signals measured in presence and absence of ligand and plotted in the wheel diagram from the different sucrose fractions (F3 to F12). They are representative of three independent experiments.

The biased activity of ligands has been assigned to their ability to stabilize distinct GPCR conformations^11^. Moreover, membrane lipids have also recently been shown to play a key role in the allosteric modulation of GPCR activity by stabilizing specific receptor conformations^33^. Also, one mechanism which could account for the biased pharmacology of β-adrenergic agonists as a function of the isoform and expression level of the Gα subunit relies on the different distribution of the Gα subunit within the plasma membrane in diverse lipid territories that could interact with different receptor populations. Hence, one ligand could promote different conformations of β-AR/G protein complexes with different outputs depending on their compartmentalization at the cell surface. We first tested this hypothesis by decoding the conformations of receptor-Gα subunit complexes in living HEK293T cells as a function of Gα expression levels using a BRET assay monitoring the direct interaction between the β1-AR receptor (β1-AR-GFP10) and the Gαi1 (Gαi1-Rluc8) subunit as previously described^10, 29^. The R-G conformations stabilized by ISO, NE or EPI were strongly dependent on Gαi1 expression (Fig. 5a). Indeed, the relationship between R-G conformations and Gαi1 expression levels was better fitted using a second order polynomial curve model than a linear one for all of the ligands (F values: 1.775, 2.932 and 7.325 for ISO, NE and EPI respectively). The difference in curvilinearity between the three ligands further confirmed their biased efficacy relative to Gα expression levels. Noteworthy, for all ligands tested, the number of R-G conformations increased considerably inversely to Gα expression levels. These results highly suggest that the different β-AR agonists recognize distinct populations of receptor-G protein complexes at the cell surface, most likely located in different membrane territories. To gain further insight into this assumption, we directly measured G protein activation in different lipid membrane territories. More specifically, low density membrane microdomains (“rafts”), the most common ones, were isolated by cell solubilization with 1% Triton X-100 at 4 °C followed by sucrose gradient separation, as previously described^34^. Thus, we measured BRET^1^ on each sucrose fraction obtained from the solubilization of HEK293T cells co-expressing β1-AR with high expression levels of Gαi1-RLuc8 in the presence of Venus-Gγ2 and Gβ3 subunits, similarly to that in Fig. 3. G protein activation triggered by the three agonists ISO, NE and EPI, was plotted in a web as a function of the sucrose fraction (F3 to F12) (Fig. 5b) and according to low (left panel) or high (right panel) relative Gα-Rluc8 expression. The webs clearly indicate that each agonist initiated a specific activation profile that was dependent not only on the fraction but also on Gα expression (Fig. 5b and Supplementary Fig. 8).

### Gα isoform and stoichiometry dictates differential β2-adrenergic receptor membrane partitioning

**Figure 6:**
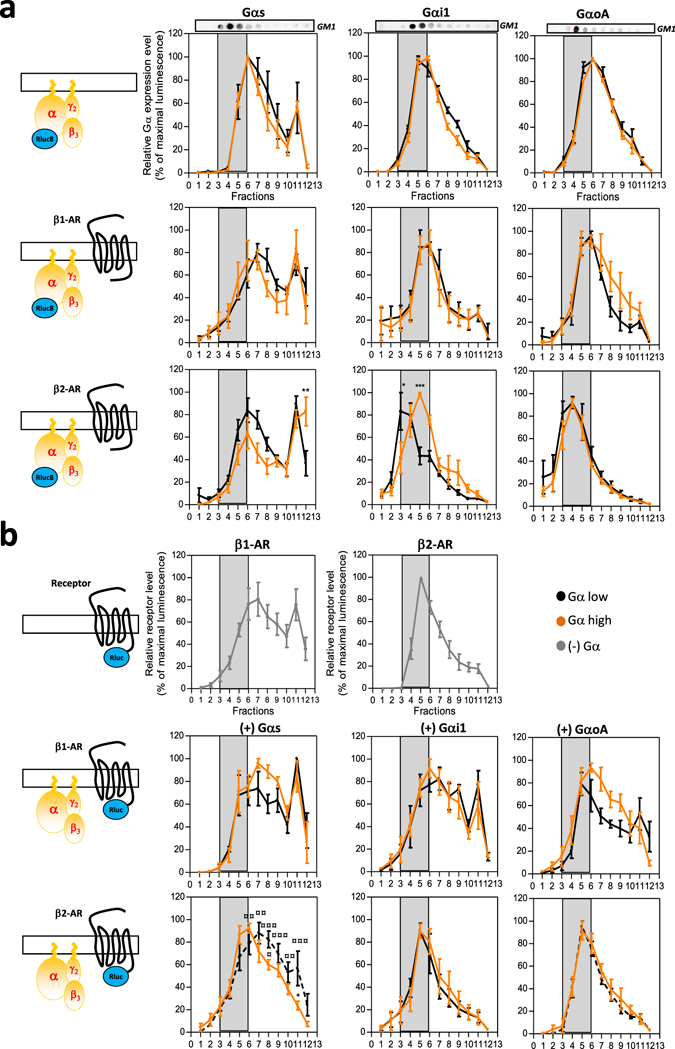
Gα expression level influences Gα subunit and β-AR receptor compartmentalization in cholesterol-enriched membranes. Detergent-resistant-membranes were purified using a triton X-100 lysis method followed by a separation on sucrose gradient from (**a**) HEK293T cells co-expressing high (orange) and low (black) expression levels of Gαs-Rluc8 (left panel), Gαi1-Rluc8 (middle panel) or GαoA-Rluc8 (right panel) along with Gβ3 and Gγ2 subunits in the presence or not (upper panels) of untagged β-AR receptors, (**b**) HEK293T cells co-expressing β1-AR-Rluc or β2-AR-Rluc alone (upper panels) or in the presence of high (orange) or low (black) expression levels of untagged Gαs (left panels), Gαi1 (middle panels) or GαoA (right panels) and Gβ3 and Gγ2 subunits. Relative receptor or Gα subunit expression levels were quantified in each sucrose fractions by recording of the total luminescence. Results are expressed as the percentage of the maximal luminescence measured from all fractions in each experiment. Grey boxes highlight raft nano-domains enriched fractions, identified by detection of the GM1 protein (upper dot plot). Data represent the mean ± s.e.m. of at least three independent experiments. The statistical significance of the difference in membrane distribution in low versus high conditions (*) or versus receptor alone (¤) was assessed using two-way ANOVA followed by a Bonferroni posttest (*P < 0.05, **P < 0.01, ***P < 0.001).

To gain further insight into the molecular mechanisms underlying such selectivity of β-adrenergic agonist responses among different membrane lipid territories depending on the Gα isoform and expression level, we finally examined the membrane partitioning of both β-AR and the Gα subunits at high and low Gα expression level conditions at resting state. HEK293T cells, co-expressing β1- or β2-AR and Gαs, Gαi1 or GαoA in the presence of Gγ2 and Gβ3 subunits similarly to that in Fig. 3, were proceeded for raft purification and we then studied the distribution of both the β1/2-AR and the Gα subunits at high or low Gα expression levels similar to the previous BRET experiments (Fig. 6). To improve detection sensitivity, we took advantage of β1/2-AR-Rluc or Gα-Rluc8 constructs whose luminescence measurements can more accurately quantify both proteins separately in each sucrose fraction. The GM1 raft marker was used as a positive control to identify raft-enriched fractions in dot-blot. Validation of luminescence recording as a valuable tool in depicting protein compartmentalization in lipid rafts isolated in a sucrose gradient, was provided by a GABAB-R2-*R*luc fusion construct encoding the prototypical neuronal R2 subunit of the GABAB receptor which usually functions in these specific microdomains^35^. As expected, when overexpressed in HEK293T cells, GABAB-R2-*R*luc was concentrated mostly in raft-enriched fractions (Fractions 2–6) at the top of the gradient but could also be detected in some non raft fractions at the extreme gradient bottom (Fractions 10–12) (Supplementary Fig. 9). When following Gα subunit distribution (Fig. 6a), each of the three subunits exhibited a specific distribution pattern. In agreement with their lipid modifications^36^, in the absence of receptor co-expression and independently of their expression levels, Gαi1 and GαoA were essentially concentrated in raft-enriched fractions while Gαs was more largely distributed in non-raft domains. Interestingly, further addition of β2-AR (Fig. 6a, lower panels) significantly modified the repartition pattern of some Gα isoforms at high expression levels, with the Gαi1 subunit more shifted in the raft domains to the detriment of the non-rafts ones while conversely Gαs expression was shifted to non-raft domains at the gradient bottom to the detriment of the rafts. Moreover, β2-AR expression shifted the entire GαoA profile to raft-concentrated domains at both high and low expression levels, decreasing GαoA expression in non-rafts. This observation is receptor-specific as different Gα gradient profiles were obtained with β1-AR (Fig. 6a, middle panels). Indeed, β1-AR expression stretched the Gαs repartition along the gradient, thus increasing its expression in both raft and non-raft domains for low and high Gαs expression levels, whereas Gαi1 had decreased expression in non-raft fractions in favor of rafts. β1-AR differently impacted on GαoA distribution according to its expression levels as it decreased GαoA in non-rafts while increasing it in rafts when considering low GαoA expression level. At high GαoA expression level, the G protein is conversely increased in non-raft fractions.

When now considering the β2-AR distribution, and as previously reported^34^, in the absence of G protein over-expression, β2-AR was enriched in raft domains (Fig. 6b, upper panels). Unexpectedly, while the distribution profiling of β2-AR was not modified by either high or low levels of GαoA expression (Fig. 6b, lower panels), in the presence of Gαs it shifted toward non-raft fractions. This could in part explain, together with the specific Gαs distribution, the modifications in cAMP production when both the β2-AR and the Gαs proteins were over-expressed (Supplementary Fig. 4). In the absence of G protein expression, β1-AR repartition was consistently more concentrated in non-raft domains compared to β2-AR (Fig. 6b, upper panels) and was less impacted by Gαs or Gαi1 expression (Fig. 6b, middle panels). However, high GαoA expression increased β1-AR expression in rafts and decreased it in non-rafts. Oddly, modification of the β-AR raft-profiling in the presence of the different Gα subunits did not necessarily correlate with the Gα profiling and vice-versa, suggesting that the profiling of the receptor co-expressed with a peculiar Gα protein most likely results from a specific interplay between the two proteins and not from the specific influence of one protein on the other.

These result highlights for the first time the impact of the Gα subunit on the receptor membrane distribution that could account for specific biased pharmacology.

## Discussion

In this study, we used controlled experimental conditions with accurate calibration of protein expression in a recombinant cell system, and have identified the importance of the stoichiometry of the G protein Gα subunit in the biased-activity of GPCR ligands. This occurs through an unexpected molecular mechanism at “basal state” of the cell governing different distribution of both the Gα subunit but also the receptor within specific membrane subdomains.

A major finding of our study is the influence of Gα subunit expression levels on both the potency and efficacy of agonist ligands at a particular receptor. It is now well accepted that a precise stoichiometry among the signaling components determines the predominant signaling response, and the influence of GPCR and G protein expression levels on ligand efficacy have been previously documented^14, 15^. Most studies have indeed extensively examined receptor stoichiometry on ligand behavior but only few data are available regarding the impact of G protein expression^18^. The influence of G protein over-expression has essentially been examined on inverse agonist efficacy at constitutive receptors and interpreted as a result of modulation of the R/R* ratio, in agreement with the predictions from the allosteric model for receptor activation (extended ternary/cubic model)^37, 38^. According to this classical model, ligand efficacy should evolve through a linear relationship with G protein expression levels, consistent with different degrees of this ligand to stabilize the R or R* receptor state as a function of the G protein level. However, although this assumption can be easily inferred from reconstituted systems^38^, it clearly differs from our results since in our living cell system with constant and saturating receptor concentrations, low and high levels of Gα subunit over-expression were non linearly correlated with ligand potency and efficacy at G protein activation and instead dictated ligand biased-behavior (Fig. 4). Interestingly, a low versus high G protein stoichiometric-dependence of ligands could not be appreciated from a downstream G protein effector, *i.e*. the adenylyl cyclase activity promoted by Gs (Supplementary Fig. 4), most probably due to the amplification and saturation of the second messenger production. Such dichotomy could also rely on experimental differences as the BRET assay specifically measured the Gα_X_β3γ2 protein complex activity while cAMP production is more global and most likely relies on a mixture of β-AR signals emanating from different G protein isoform combinations.

Indeed, the GPCR activation theory mainly assumes a free receptor/G protein/effector system in a fluid mosaic model where the membrane is considered a homogeneous lipid medium. However, biological membranes are much more complex and harbor compartments with different component partitions, thus introducing the notion of a spatial signaling constraint. GPCRs and G proteins, as inserted membrane proteins, do not escape this rule. In a mammalian cell, each of them are subjected to different lipid post-translational modifications^36, 39^ that dictate particular membrane subdomain localizations and activities. In this study, we demonstrated that the degree of Gα subunit expression level differently modulates the efficacy/potency of a ligand based on different partitioning of both the G protein and the receptor, at least in part, in raft-enriched domains. In agreement with microdomain-dependent ligand efficacy, we found that the G protein level stabilizes different conformations of the receptor-Gα complex. This observation completely reconciles the ligand-biased-behavior that until now was inferred from the stabilization of different receptor conformations^11^. Several studies have already reported the different influences of the lipid membrane composition on receptor pharmacology^33, 40^ and specifically cholesterol enriched in raft domains was recently identified as a key trigger that can directly bind and activate GPCRs of GPCR^41^. Although each Gα protein subunit demonstrates a specific subdomain distribution^36^, the fact that modification of the Gα subunit level can modify the membrane distribution of G protein is not surprising and could be easily reconciled with its overexpression resulting in saturation of certain cell compartments with consequent occupation of new ones. Accordingly, endogenous adenylyl cyclase 6 was shown to be localized primarily in caveolae-rich domains in rat aortic smooth muscle cells while overexpressed AC6 was localized only in non-caveolae domains^42^. More outstanding finding is the impact of the Gα expression on the receptor partitioning at resting state since receptor shuttling in different membrane domains has been mostly reported upon agonist stimulation^43, 44^. Overall, this result could indeed refer to the existence of a preformed receptor-G protein complex that several groups have already described^45–48^. In this model, the biochemical properties of both the receptor and the G protein in the complex could differ from each individual protein taken separately and thus dictate specific trafficking-partitioning properties depending on the G protein and the receptor species as shown by our results. In line with this hypothesis, despite the presence of palmitoylation lipid modifications, some proteins like CD4 do not necessarily require palmitoylation for lipid raft localization^49^. Finally, in agreement with this model, Scarlata *et al*. previously suggested a role for Gq in directing the bradykinin type 2 receptor into caveolae domains^50^.

In addition to the G protein stoichiometry parameter, in this study we have also confirmed the influence of the G protein isoform on the functional selectivity of ligands, as recently outlined^12, 13^. From this standpoint, an important finding is that the three β-AR agonists, ISO, NE and EPI, behaved as biased agonists relative to each other, as reflected by their differences in potencies/efficacies on the activation of the three G proteins tested, namely Gαs, GαoA and Gαi1 (Fig. 4). This result corroborates that of Lohse’s group who demonstrated that these agonists stabilize different β2-AR conformations^51^. Hence, ISO, NE and EPI most probably promote selective signaling pathways and cellular responses at β-ARs with different physio/pathophysiological responses. These data should have major impact on the β-adrenergic field in which ISO is usually taken as a more stable surrogate agonist for natural NE /EPI especially when exploring the physio/pathophysiology of β-AR-dependent cardiovascular regulation. It therefore follows that previous conclusions obtained with ISO to make an assumption on NE or EPI effects in physio/pathophysiology should be reconsidered.

From the cardiology field standpoint, we have shown that all Gα subunits are subjected to specific important gene expression regulation in cardiomyocytes under pathological situation such as barometric-induced failure. This contrasts with several studies reporting the specific downregulation of Gαi2^23^ or Gαi1^25^ in patients with heart failure. Although this discrepancy could rely on the specificity of the phenotype, it could also result from an experimental issue since these studies examined gene expression in whole heart samples harboring different cardiac cell types and not in isolated cardiomyocytes. It will thus be interesting in the future to test whether different cardiac diseases dictate a specific fingerprint of Gα gene expression. Another interesting finding from our study is the specific downregulation of Gαi1 in the context of barometric cardiac stress, which correlated in heterologous system with a gain in its coupling to the β-AR and which could therefore account for the Gs to Gi signaling switch of the β2-AR that has been reported in pathological situations^52^. Finally, given that modification of β-AR signaling has clearly been established as a molecular contributor to the evolution of heart failure, changes in the G protein and the receptor ratio could participate in the atypical pharmacology of β-AR agonists and the onset and/or worsening of the disease. Although this assumption could also be extended to β-antagonists, an interesting finding from our study is the lower dependence to the Gα stoichiometry of metoprolol and bisoprolol, two β-blockers currently used in the management of chronic heart failure improving symptoms and reducing mortality. This is an intriguing finding when considering our initial results showing high fluctuations in Gα gene expression levels in failing cardiomyocytes. Indeed, on one hand, preserved efficacy of β-blockers across different Gα expression levels could account, at least in part, for their clinical efficacy but also for the lack of clear clinical advantage of one drug over the other^53^. On the other hand, the high variability of responses to β-blockers compared to β-agonists in our experiments, especially in the low Gαi1 and GαοΑ level range, could also refer to the still unpredictable 30% of non-responders to β-blockers in clinical trials. Altogether, these results further reinforce the recent notion of dynamic bias and its implication for GPCR drug d iscovery^54^.

In summary, the present study highlights the importance of G protein stoichiometry as an important contributor to the bias of ligands. Thus, the biased activity of ligands should be considered when comparing cells with similar G protein repertoires. However, the stoichiometry of the other signaling partners will most probably affect the bias of ligands as well. This truly questions the relevance of bias signaling analysis/quantification in recombinant systems that have been used as standard up to now when comparing different signaling pathways with variable manipulation of receptor and effector expression levels (G protein, β-arrestin, GRK…)^19^. From an industrial standpoint, our results could help fine tuning the assays used in HTS programs to increase the yield of positive hits for biased ligand identification. However, even with the most biased hit ligand, its exact bias translation in *in vivo* systems with varying cell types and signaling components will still remain a tall order and will not necessarily predict its therapeutic efficacy. Thus, the *in vitro* identification of biased ligands does not have to ring as a predictive therapeutic value but more as a yield component and all molecule hits obtained from this *in vitro* screen will have to be systematically tested *in vivo* without *a priori* and without shortlisting.

## Methods

### Live animals

Experimental animal protocols were carried out in two month old male C57BL/6 mice in accordance with the French regulation guidelines for animal experimentation and were approved by the CEEA-122 ethical committee.

### Materials

(−)-Isoproterenol hydrochloride (ISO), (−)-norepinephrine (+) bitartrate salt monohydrate (NE), (−)-epinephrine (EPI), and angiotensine II (AngII) were purchased from Sigma-Aldrich (St. Louis, MO, USA) while coelenterazine *400a* and coelenterazine *h* were purchased from Interchim.

### Quantification of Gα subunits gene expression

Pathological cardiac hypertrophy was induced in 6-week-old male C57BL/6 J mice by transversal aortic banding (TAC). Animals were analyzed 15 days after TAC and their cardiac characterization was previously reported^28^. Fifteen days after TAC, cardiomyocytes were then isolated and total RNA isolation and real-time quantitative RT-PCR were conducted as previously described^28^. Relative gene expression of Gα subunits in sham versus TAC mice were calculated using the comparative *Ct* method after quantitative PCR performed using the Fluidigm Biomark HD nano-scale platform. Genes encoding the Glyceraldehyde-3-phosphate dehydrogenase (GAPDH) and the Hypoxanthine-guanine phosphoribosyltransferase (HPRT) were used as housekeeping genes for normalization. Primers for detection of murine Gα subunits genes were provided and validated by Fluidigm (DELTAgene^TM^ assays).

### cDNA expression vectors

Plasmids encoding HA-AT_1A_-R, HA-β2-AR, β1-AR-GFP10, β2-AR-Rluc, HA-β1-AR, β1-AR-Rluc, Gαs-Rluc8, Gαi1-Rluc8, GαoA-Rluc8 and GFP10-Gγ_2_ were previously described^10, 46^. Gβ3 encoding vector was obtained from the Missouri S&T cDNA Resource Center.

### Cell culture and transfection

Human embryonic kidney 293 cells (HEK293T) cells were cultured in DMEM Glutamax supplemented with 10% (v/v) FBS and 100 units/ml penicillin/streptomycin at 37 °C in a humidified atmosphere at 5% CO2. Transient transfections were performed 24 h after cell seeding using polyethylenimine (PEI, Polysciences Inc.).

### Bioluminescence resonance energy transfer (BRET) measurements

Receptor and G proteins subunit-encoding vectors were transiently transfected into HEK293T cells as indicated in the figure legends. Forty-eight hours after transfection, cells were washed with PBS, detached in PBS/5 mM EDTA and resuspended in PBS/0.1% (w/v) glucose at room temperature. Cells were then distributed (80 µg of protein per well) into a 96-well microplate (Wallac, PerkinElmer Life and Analytical Sciences) and incubated in the presence of the different ligands for 1 min. BRET^2^ between Rluc8 and GFP10 was measured after the addition of the Rluc8 substrate coelenterazine *400a* (5 µM, Interchim). BRET readings were collected using a modified Infinite F500 (Tecan Group Ltd). The BRET^2^ signal was calculated by the ratio of GFP10 emission (510–540 nm) to that of RLuc/Rluc8 (370–450 nm).

### Biochemical purification of cholesterol-enriched microdomains

Receptor and G proteins were transiently transfected in HEK293T cells as indicated in the figure legends. Detergent-resistant membrane microdomains isolation was conducted 48 hours after transfection as previously described^34^. Briefly, cells were washed, detached in cold PBS and solubilized in ice-cold lysis buffer (Tris 25 mM pH 7.4; NaCl 140 mM; EDTA 2 mM) containing 1% Triton × 100 (v/v) (Sigma) and a protease inhibitor cocktail (Complete mini; Roche, Bale, Switzerland). Lysates were then homogenized and centrifuged 10 min at 860 g at 4 °C. Supernatants were collected and protein content was evaluated using the BioRad DC^TM^ protein assay. An equal amount of proteins for each individual transfection was then adjusted to 60% sucrose and placed at the bottom of an Ultra-Clear centrifuge tube (Beckman instruments), and overlaid with a 5–35% discontinuous sucrose gradient prepared in the lysis buffer (without triton). Gradients were centrifuged at 39 000 rpm for 18 h at 4 °C without breakdown in a SW41 rotor (Beckman L70 Ultracentrifuge). Then, twelve 1 ml fractions were collected from the top of the gradients. Total luminescence in each fraction (180 µl) was measured in a 96-well microplate following addition of 5 µM of the luciferase substrate, coelenterazine *h*, using a luminometer Mithras LB 940 (Berthold technologies, Germany). GM1 gangliosides, a marker of the raft microdomains, was detected in each fraction by dot plot using peroxydase-coupled cholera toxin subunit B conjugates (Molecular probes). Sucrose density was controlled by refractometry.

### Quantification of cell surface receptors by ELISA

HA-tagged receptors and G proteins were transiently transfected in HEK293T cells as indicated in the figure legends. Twenty-four hours after transfection, cells were splited in 24-well plates precoated with poly-L-lysine. The next day, cells were fixed in 4% paraformaldehyde, saturated with PBS containing 1% bovine serum albumin and incubated with the primary anti-HA antibody (clone 16B12; Covance) and then with the HRP-labeled secondary antibody (Sigma, St. Louis, MO, USA). After washing, cells were incubated with HRP substrate TMB (3,39,5,59-tetramethylbenzidine; BD Biosciences). The reaction was stopped with HCl 1 N and the plates were read at 450 nm in a microplate reader (Varioscan Flash, Thermo Electron). The 570 nm optic density (background) was subtracted according to the manufacturer.

### Western blot analysis

Receptor and G proteins were transiently transfected in HEK293T cells as indicated in the figure legends. For Gα protein expression level analysis, cells were lysed in a lysis buffer containing 50 mM Tris pH 7.4, 150 mM NaCl, 1 mM EDTA, 1% triton, H2O and cocktail protease inhibitors (ROCHE). Cells lysates (50 µg) were subjected to SDS-PAGE electrophoresis under reducing conditions, transferred onto PVDF membranes (Millipore), and analyzed by immunoblotting according to standard protocols using anti-Gαs polyclonal primary antibody recognizing Gα sequence specifically outside of the Rluc8 insertion site (PA5-19315; Thermo fisher Scientific), and an anti-GAPDH monoclonal antibody (sc-47724; Santa Cruz Biotechnology). Rabbit anti-Goat and Sheep anti-Mouse HRP-labeled secondary antibodies were from Thermo fisher Scientific (31402) and GE Healthcare (NA931V) respectively.

### cAMP quantification

Quantification of cAMP levels was performed using the HTRF assay (Homogeneous Time-Resolved Fluorescence): dynamic2 cAMP kit (Cisbio, Bedford, USA), based on a competitive immunoassay using Lumi4-TbTM cryptate-labeled antibodies anti-cAMP and d2-labeled cAMP. For that purpose, HEK293T cells were transiently transfected as indicated in the figure legends and 48 h post-transfection, cells were washed and resuspended in PBS/5 mM Glucose/2 mM IBMX. Then, 10,000 cells/well (384 wells-plate) were plated and stimulated for 1 h at room temperature with increasing ligand concentrations in a final volume of 10 µL. Cells were then lysed using 5 µL of lysis buffer containing d2-labeled cAMP and 5 µL of Lumi4-TbTM cryptate-labeled anti-cAMP. The signal was measured after 1 h room temperature incubation using a modified Infinite F500 (Tecan Group Ltd). The RET signal was calculated by the ratio of d2-cAMP/Lumi4-TbTM (665 nm/620 nm), the specific signal being inversely proportional to the concentration of cAMP in the sample. For each experiment, a calibration curve was established with cAMP standards allowing the quantification of cAMP levels by linear regression.

### Data and statistical analysis

Statistical analysis of genomic changes between sham and TAC cardiomyocytes and BRET signal modulations were carried out using GraphPad Prism 4 software (GraphPad Software Inc., San Diego, CA). Statistical tests used are indicated in the figure legends. Values of *P* < 0.05 were considered statistically significant.

## Electronic supplementary material

Supplementary Information

## References

[1] Kenakin T (2011). Functional selectivity and biased receptor signaling. J Pharmacol Exp Ther.

[2] Kingwell K (2015). Pioneering biased ligand offers efficacy with reduced on-target toxicity. Nat Rev Drug Discov.

[3] Patel CB, Noor N, Rockman HA (2010). Functional selectivity in adrenergic and angiotensin signaling systems. Mol Pharmacol.

[4] Kubon C (2011). The role of beta-blockers in the treatment of chronic heart failure. Trends Pharmacol Sci.

[5] Poole-Wilson PA (2003). Comparison of carvedilol and metoprolol on clinical outcomes in patients with chronic heart failure in the Carvedilol Or Metoprolol European Trial (COMET): randomised controlled trial. Lancet.

[6] Thanawala VJ (2014). Ligand bias prevents class equality among beta-blockers. Curr Opin Pharmacol.

[7] Masri B (2008). Antagonism of dopamine D2 receptor/beta-arrestin 2 interaction is a common property of clinically effective antipsychotics. Proc Natl Acad Sci USA.

[8] Allen JA (2011). Discovery of beta-arrestin-biased dopamine D2 ligands for probing signal transduction pathways essential for antipsychotic efficacy. Proc Natl Acad Sci USA.

[9] Wei H (2003). Independent beta-arrestin 2 and G protein-mediated pathways for angiotensin II activation of extracellular signal-regulated kinases 1 and 2. Proc Natl Acad Sci USA.

[10] Sauliere A (2012). Deciphering biased-agonism complexity reveals a new active AT1 receptor entity. Nat Chem Biol.

[11] Shukla AK (2008). Distinct conformational changes in beta-arrestin report biased agonism at seven-transmembrane receptors. Proc Natl Acad Sci USA.

[12] Klein Herenbrink C (2016). The role of kinetic context in apparent biased agonism at GPCRs. Nat Commun.

[13] Masuho I (2015). Distinct profiles of functional discrimination among G proteins determine the actions of G protein-coupled receptors. Sci Signal.

[14] Kenakin T (1997). Differences between natural and recombinant G protein-coupled receptor systems with varying receptor/G protein stoichiometry. Trends Pharmacol Sci.

[15] Kenakin T (2002). Efficacy at G-protein-coupled receptors. Nat Rev Drug Discov.

[16] Kenakin T (2015). Gaddum Memorial Lecture 2014: receptors as an evolving concept: from switches to biased microprocessors. Br J Pharmacol.

[17] Kenakin TP (2016). Synoptic pharmacology: Detecting and assessing the pharmacological significance of ligands for orphan receptors. Pharmacol Res.

[18] Watson C (2000). The use of stimulus-biased assay systems to detect agonist-specific receptor active states: implications for the trafficking of receptor stimulus by agonists. Mol Pharmacol.

[19] Galandrin S, Onfroy L, Poirot MC, Senard JM, Gales C (2016). Delineating biased ligand efficacy at 7TM receptors from an experimental perspective. Int J Biochem Cell Biol.

[20] Doi M (2016). Gpr176 is a Gz-linked orphan G-protein-coupled receptor that sets the pace of circadian behaviour. Nat Commun.

[21] Grant KR, Harnett W, Milligan G, Harnett MM (1997). Differential G-protein expression during B- and T-cell development. Immunology.

[22] Chang GW (2007). CD312, the human adhesion-GPCR EMR2, is differentially expressed during differentiation, maturation, and activation of myeloid cells. Biochem Biophys Res Commun.

[23] Eschenhagen T (1992). Increased messenger RNA level of the inhibitory G protein alpha subunit Gi alpha-2 in human end-stage heart failure. Circ Res.

[24] Ishikawa Y (1994). Downregulation of adenylylcyclase types V and VI mRNA levels in pacing-induced heart failure in dogs. J Clin Invest.

[25] Longabaugh JP, Vatner DE, Vatner SF, Homcy CJ (1988). Decreased stimulatory guanosine triphosphate binding protein in dogs with pressure-overload left ventricular failure. J Clin Invest.

[26] Yajima I (2012). Reduced GNG2 expression levels in mouse malignant melanomas and human melanoma cell lines. Am J Cancer Res.

[27] Corvol JC (2004). Persistent increase in olfactory type G-protein alpha subunit levels may underlie D1 receptor functional hypersensitivity in Parkinson disease. J Neurosci.

[28] Genet G (2012). Ephrin-B1 is a novel specific component of the lateral membrane of the cardiomyocyte and is essential for the stability of cardiac tissue architecture cohesion. Circ Res.

[29] Bellot M (2015). Dual agonist occupancy of AT1-R-alpha2C-AR heterodimers results in atypical Gs-PKA signaling. Nat Chem Biol.

[30] Rybin VO, Steinberg SF (2008). G protein betagamma dimer expression in cardiomyocytes: developmental acquisition of Gbeta3. Biochem Biophys Res Commun.

[31] Urizar E (2011). CODA-RET reveals functional selectivity as a result of GPCR heteromerization. Nat Chem Biol.

[32] M’Kadmi C (2015). Agonism, Antagonism, and Inverse Agonism Bias at the Ghrelin Receptor Signaling. J Biol Chem.

[33] Dawaliby R (2016). Allosteric regulation of G protein-coupled receptor activity by phospholipids. Nat Chem Biol.

[34] Pontier SM (2008). Cholesterol-dependent separation of the beta2-adrenergic receptor from its partners determines signaling efficacy: insight into nanoscale organization of signal transduction. J Biol Chem.

[35] Becher A, White JH, McIlhinney RA (2001). The gamma-aminobutyric acid receptor B, but not the metabotropic glutamate receptor type-1, associates with lipid rafts in the rat cerebellum. J Neurochem.

[36] Wedegaertner PB, Wilson PT, Bourne HR (1995). Lipid modifications of trimeric G proteins. J Biol Chem.

[37] Azzi M (2001). Allosteric effects of G protein overexpression on the binding of beta-adrenergic ligands with distinct inverse efficacies. Mol Pharmacol.

[38] Yao XJ (2009). The effect of ligand efficacy on the formation and stability of a GPCR-G protein complex. Proc Natl Acad Sci USA.

[39] Qanbar R, Bouvier M (2003). Role of palmitoylation/depalmitoylation reactions in G-protein-coupled receptor function. Pharmacol Ther.

[40] Zocher M, Zhang C, Rasmussen SG, Kobilka BK, Muller DJ (2012). Cholesterol increases kinetic, energetic, and mechanical stability of the human beta2-adrenergic receptor. Proc Natl Acad Sci USA.

[41] Huang P (2016). Cellular Cholesterol Directly Activates Smoothened in Hedgehog Signaling. Cell.

[42] Ostrom RS (2002). Localization of adenylyl cyclase isoforms and G protein-coupled receptors in vascular smooth muscle cells: expression in caveolin-rich and noncaveolin domains. Mol Pharmacol.

[43] Pike LJ (2003). Lipid rafts: bringing order to chaos. J Lipid Res.

[44] Rybin VO, Xu X, Lisanti MP, Steinberg SF (2000). Differential targeting of beta -adrenergic receptor subtypes and adenylyl cyclase to cardiomyocyte caveolae. A mechanism to functionally regulate the cAMP signaling pathway. J Biol Chem.

[45] Damian M (2015). Ghrelin receptor conformational dynamics regulate the transition from a preassembled to an active receptor:Gq complex. Proc Natl Acad Sci USA.

[46] Gales C (2006). Probing the activation-promoted structural rearrangements in preassembled receptor-G protein complexes. Nat Struct Mol Biol.

[47] Nobles M, Benians A, Tinker A (2005). Heterotrimeric G proteins precouple with G protein-coupled receptors in living cells. Proc Natl Acad Sci USA.

[48] Qin K, Dong C, Wu G, Lambert NA (2011). Inactive-state preassembly of G(q)-coupled receptors and G(q) heterotrimers. Nat Chem Biol.

[49] Popik W, Alce TM (2004). CD4 receptor localized to non-raft membrane microdomains supports HIV-1 entry. Identification of a novel raft localization marker in CD4. J Biol Chem.

[50] Calizo RC, Scarlata S (2012). A role for G-proteins in directing G-protein-coupled receptor-caveolae localization. Biochemistry.

[51] Reiner S, Ambrosio M, Hoffmann C, Lohse MJ (2010). Differential signaling of the endogenous agonists at the beta2-adrenergic receptor. J Biol Chem.

[52] Gong H (2002). Specific beta(2)AR blocker ICI 118,551 actively decreases contraction through a G(i)-coupled form of the beta(2)AR in myocytes from failing human heart. Circulation.

[53] Barrese V, Taglialatela M (2013). New advances in beta-blocker therapy in heart failure. Front Physiol.

[54] Michel MC, Seifert R, Bond RA (2014). Dynamic bias and its implications for GPCR drug discovery. Nat Rev Drug Discov.

