# Supplementary material for: G protein stoichiometry dictates biased agonism through distinct receptor-G protein partitioning
Source: Sci Rep. 2017 Aug 11;7:7885. doi: 10.1038/s41598-017-07392-5 (PMC5554226; doi:10.1038/s41598-017-07392-5)
Supplement: Supplementary file 1 — Supplementary Information [file 41598_2017_7392_MOESM1_ESM.pdf]

G protein stoichiometry dictates biased agonism through distinct receptor-G protein partitioning

Lauriane Onfroy[1], Ségolène Galandrin[1], Stéphanie M. Pontier[2], Marie-Hélène Seguelas[1], Du N'Guyen[1], Jean-Michel Sénard[1,3], Céline Galés[1].

[1] Institut des Maladies Métaboliques et Cardiovasculaires, Institut National de la Santé et de la Recherche Médicale, U1048, Université de Toulouse, F-31432 Toulouse, France.

[2] Independent Researcher Ottawa, ON, Canada.

[3] Service de Pharmacologie Clinique, Centre Hospitalier Universitaire de Toulouse, Faculté de Médecine, Université de Toulouse, F-31000 Toulouse, France.

SUPPLEMENTARY FIGURES

Supplementary Figure 1

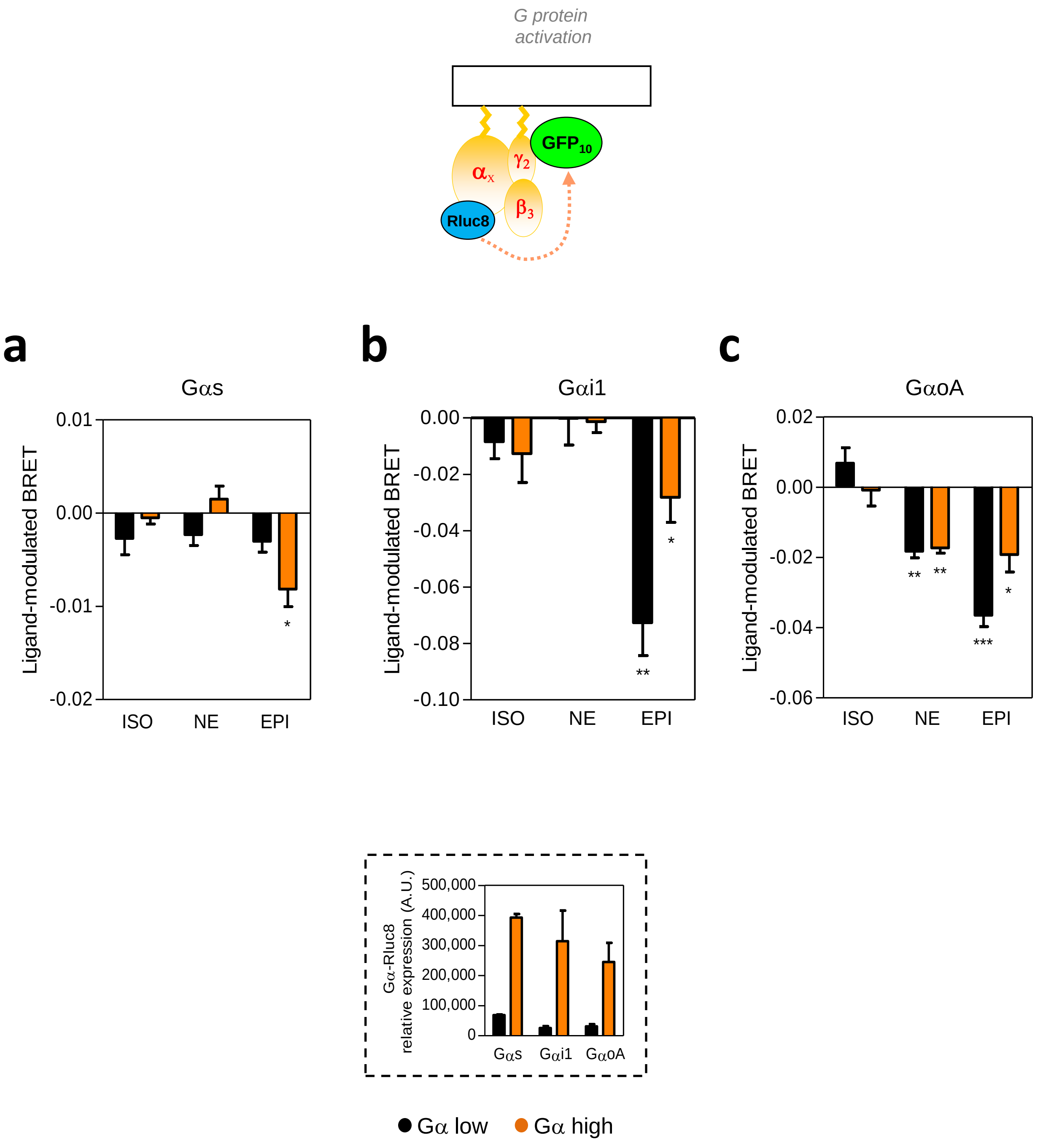

Supplementary Figure 2

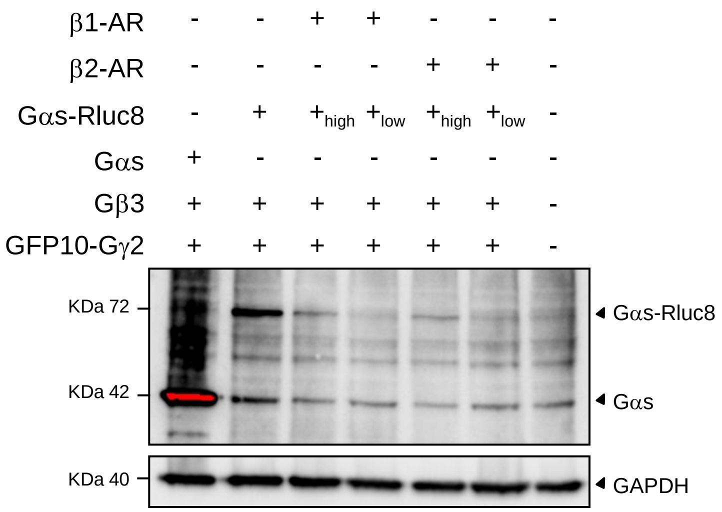

Supplementary Figure 3

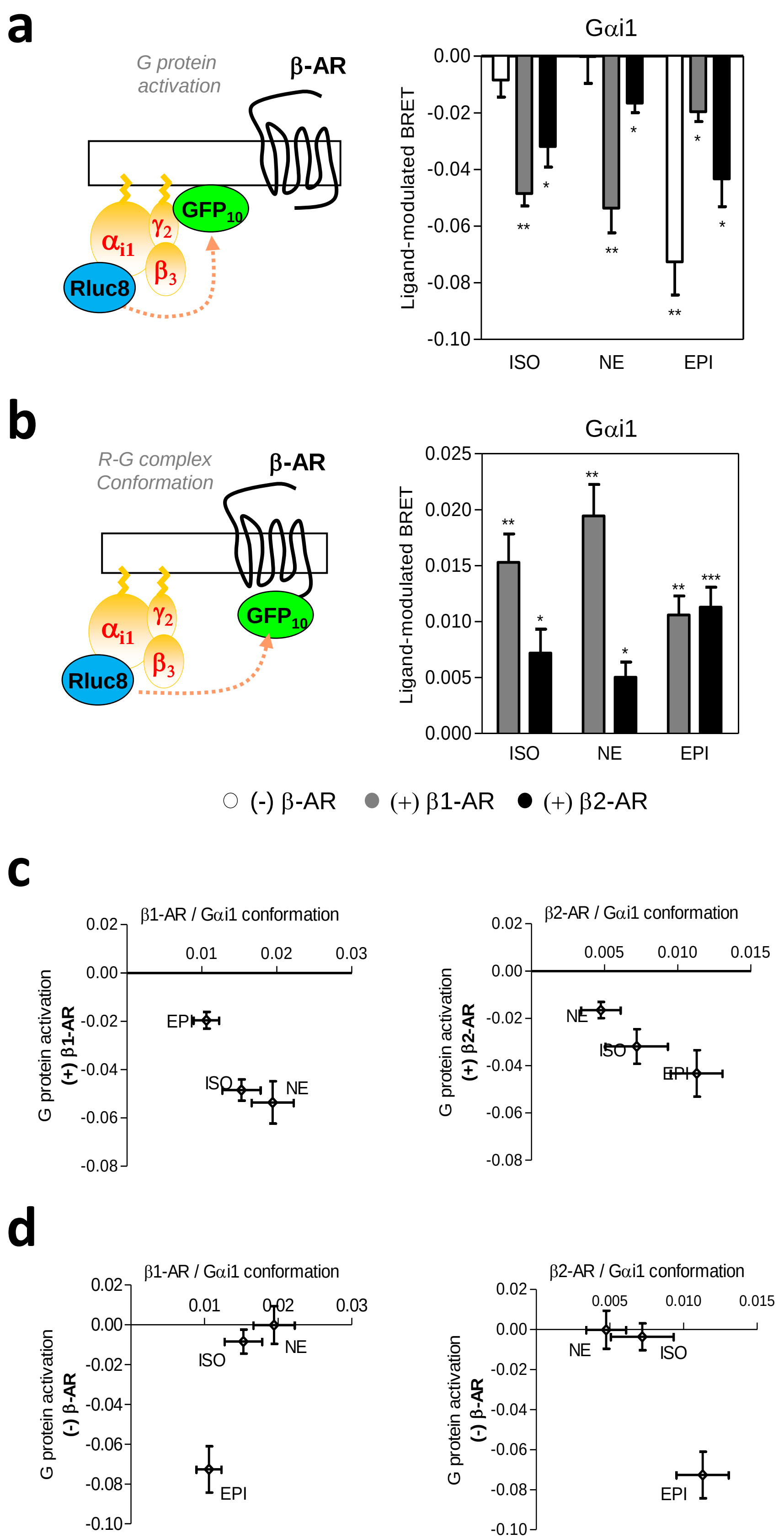

Supplementary Figure 4

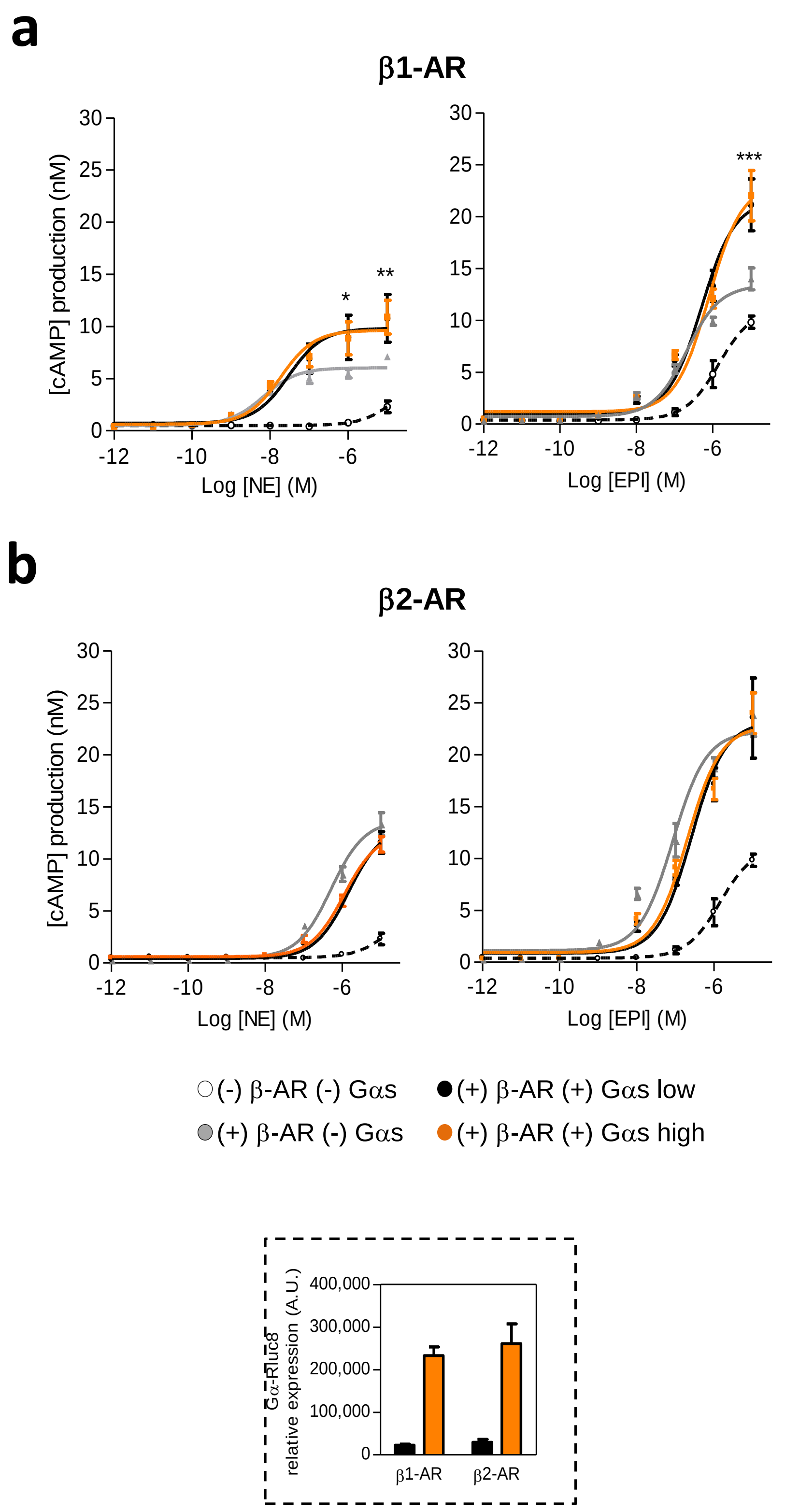

Supplementary Figure 5

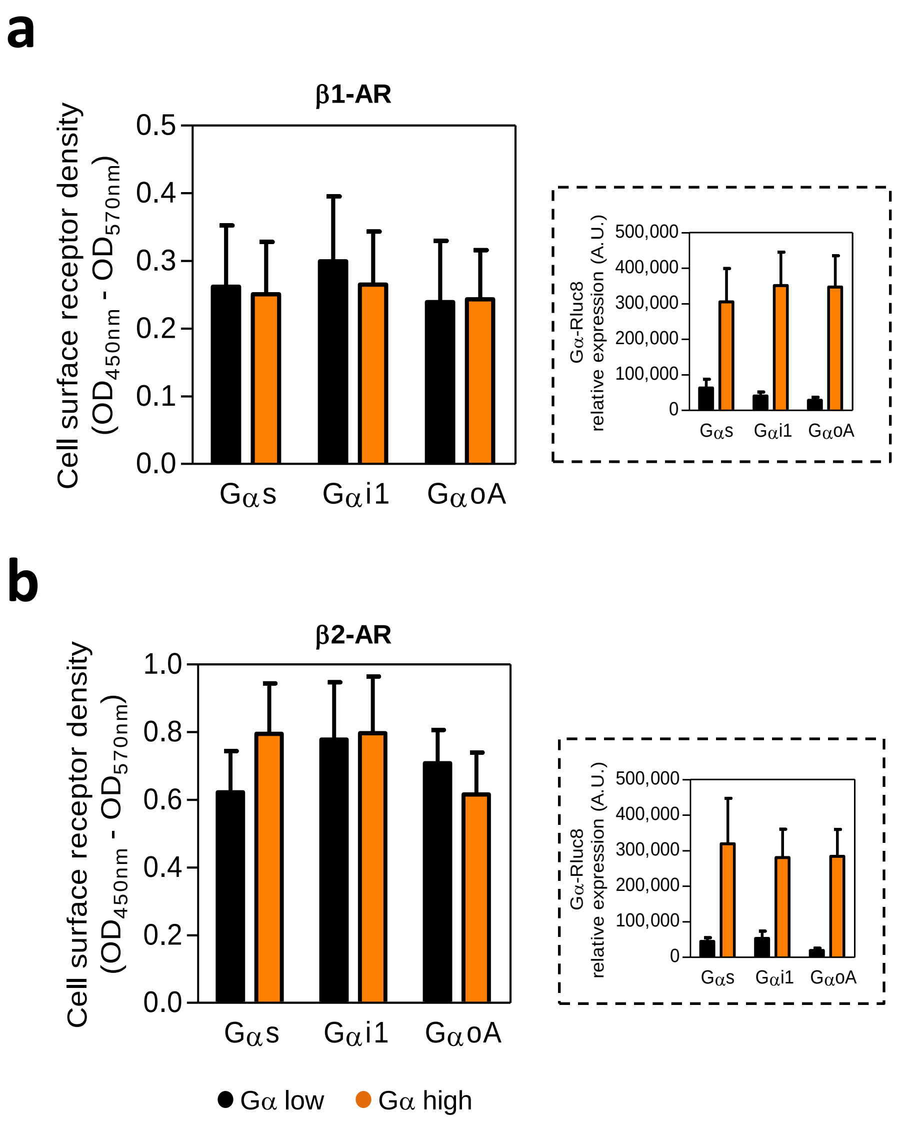

Supplementary Figure 6

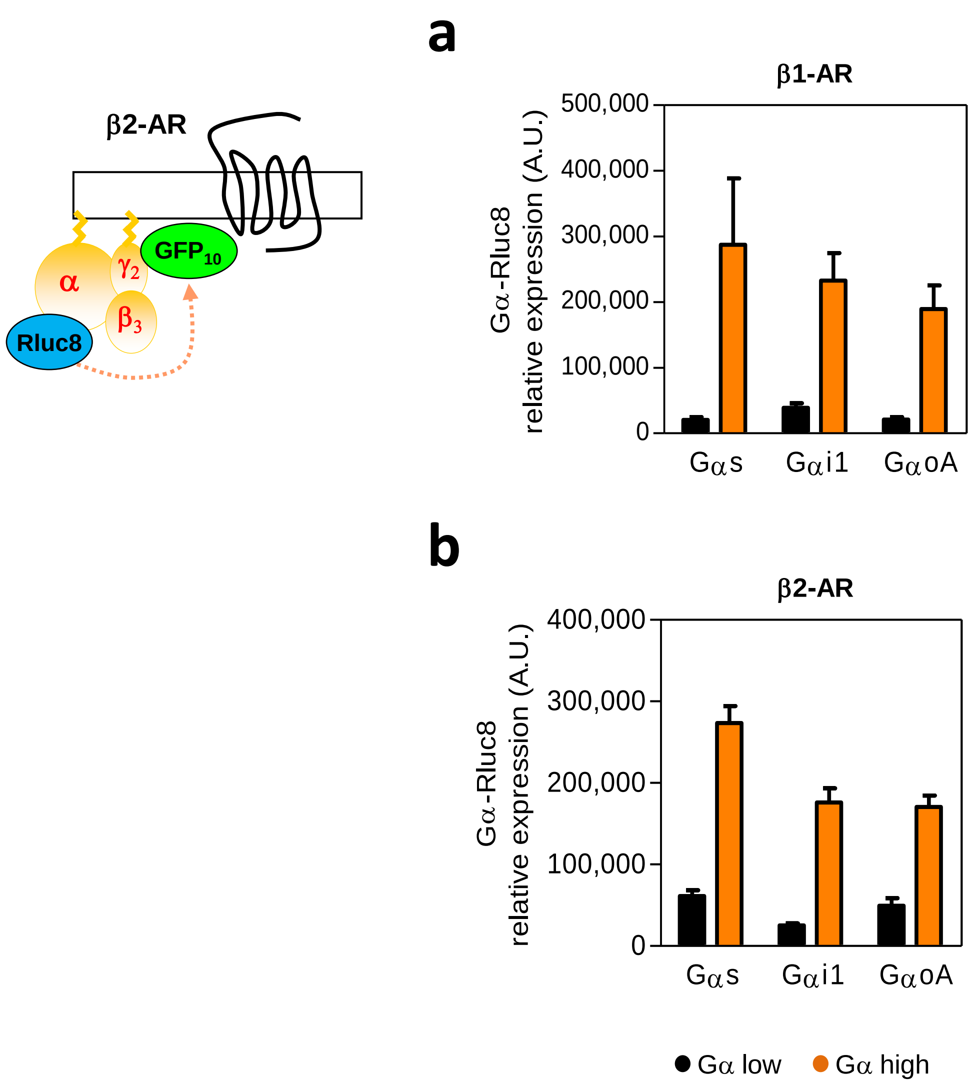

Supplementary Figure 7

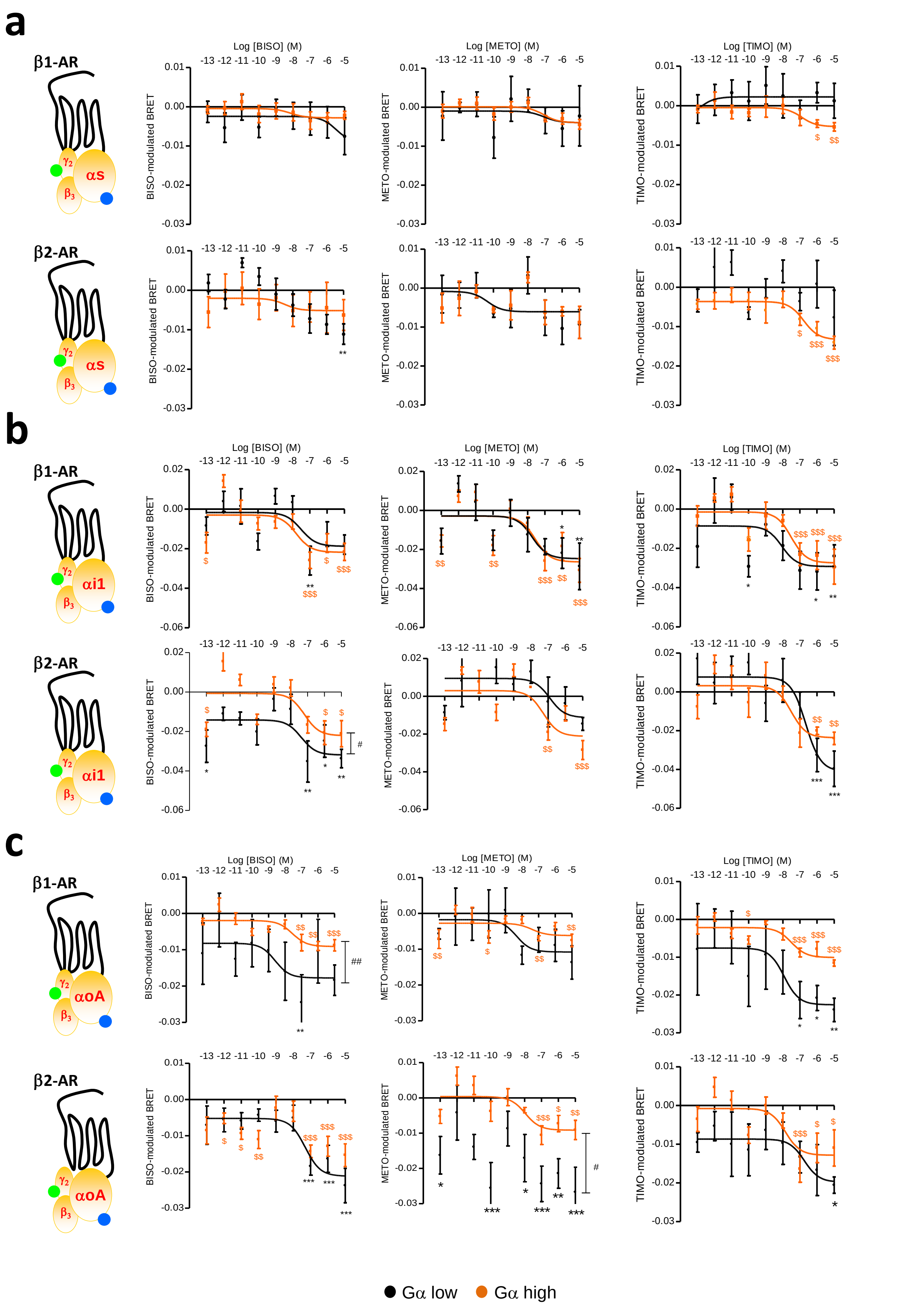

Supplementary Figure 8

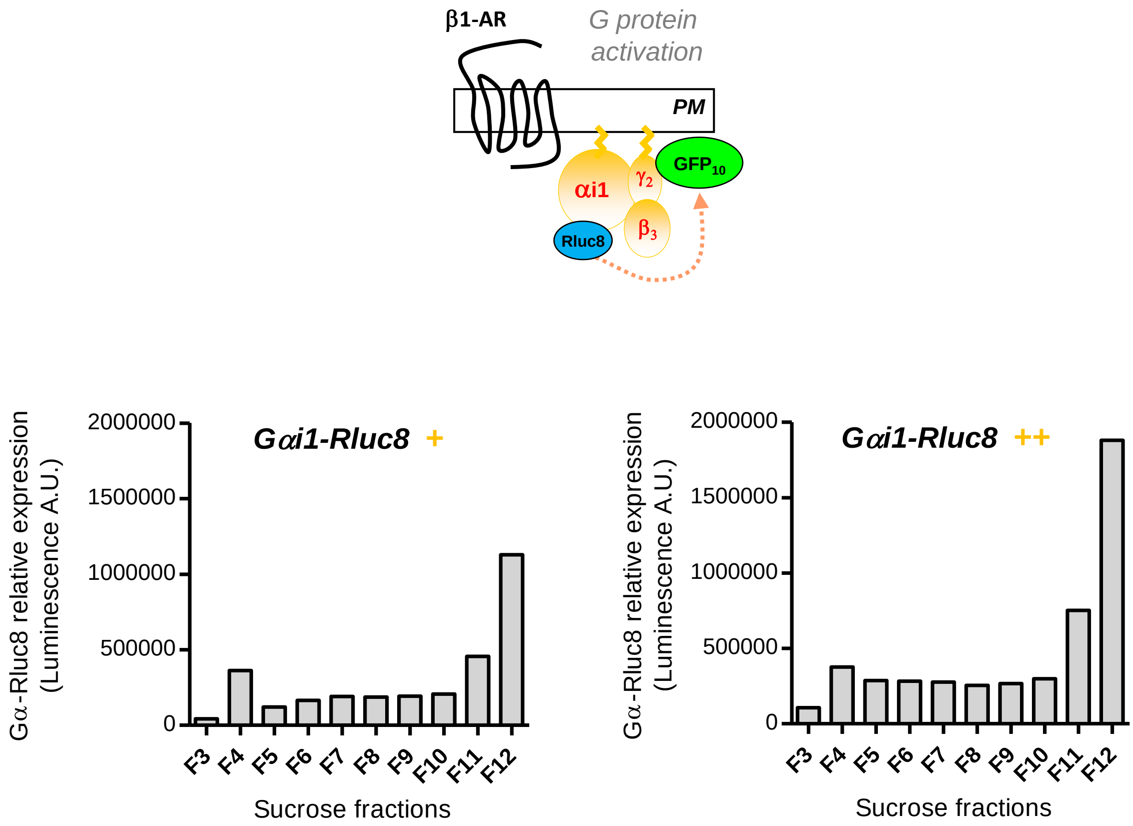

Supplementary Figure 9

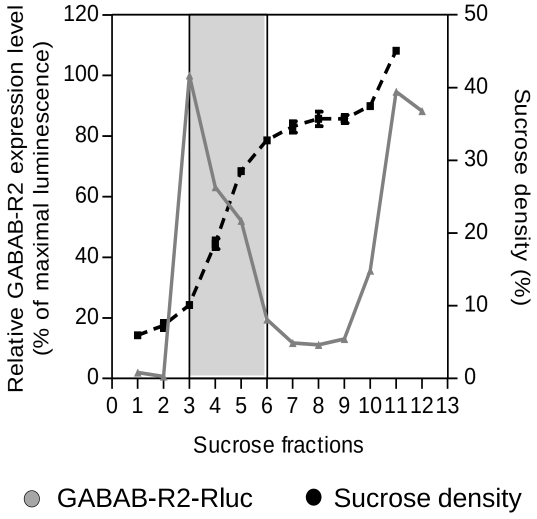

SUPPLEMENTARY TABLES

Supplementary Table 1

Ligands		NE		EPI	
(-) R<br>(-) G	**EC50**	1.63e-005		1.44e-006	
	**pEC50 ± s.e.m.**	-4.78 ± 1.14		-5.84 ± 0.10	
	**Emax ± s.e.m.**	5.25 ± 7.81		11.18 ± 0.704	
**Receptor (R)**		**β1-AR**	**β2-AR**	**β1-AR**	**β2-AR**
(+) R<br>(-) G	**EC50**	6.71e-009	5.09e-007	2.08e-007	8.53e-008
	**pEC50 ± s.e.m.**	-8.17 ± 0.13	-6.29 ± 0.08	-6.68 ± 0.10	-7.07 ± 0.11
	**Emax ± s.e.m.**	6.031 ± 0.24	13.70 ± 0.54	13.39 ± 0.56	22.25 ± 1.02
(+) R<br>(+) Gαs low	**EC50**	2.98e-008	1.45e-006	5.11e-007	2.57e-007
	**pEC50 ± s.e.m.**	-7.52 ± 0.23	-5.84 ± 0.07	-6.29 ± 0.11	-6.59 ± 0.13
	**Emax ± s.e.m.**	9.81 ± 0.76	13.15 ± 0.56	21.58 ± 1.20	23.18 ± 1.34
(+) R<br>(+) Gαs high	**EC50**	1.85e-008	1.12e-006	7.85e-007	2.02e-007
	**pEC50 ± s.e.m.**	-7.73 ± 0.18	-5.95 ± 0.07	-6.10 ± 0.11	-6.69 ± 0.09
	**Emax ± s.e.m.**	9.63 ± 0.56	12.53 ± 0.5	23.17 ± 1.31	22.79 ± 0.90

Supplementary Table 2

β1-AR

Gαs	Low			High			Low vs. High	
	**EC50**	**pEC50 ± s.e.m.**	**Emax ± s.e.m.**	**EC50**	**pEC50 ± s.e.m.**	**Emax ± s.e.m.**	**EC50**	**Emax**
**ISO**	3.61e-008	-7.44 ± 0.86	-0.009 ± 0.001	3.02e-008	-7.52 ± 0.38	-0.009 ± 0.001	ns	ns
**NE**	1.13e-008	-7.94 ± 0.61	-0.011 ± 0.001	1.25e-008	-7.90 ± 0.28	-0.008 ± 0.001	ns	0.03*
**EPI**	3.10e-006	-5.50 ± 0.53	-0.014 ± 0.005	4.65e-008	-7.33 ± 0.26	-0.012 ± 0.001	0.01**	ns
**Gαi1**	**Low**			**High**			**Low vs. High**	
	**EC50**	**pEC50 ± s.e.m.**	**Emax ± s.e.m.**	**EC50**	**pEC50 ± s.e.m.**	**Emax ± s.e.m.**	**EC50**	**Emax**
**ISO**	1.01e-007	-6.99 ± 0.67	-0.044 ± 0.007	na	na	na	ns	ns
**NE**	6.09e-008	-7.21 ± 0.57	-0.024 ± 0.005	5.27e-008	-7.27 ± 0.33	-0.039 ± 0.004	ns	ns
**EPI**	5.84e-008	-7.23 ± 0.77	-0.030 ± 0.008	na	na	na	ns	ns
**GαoA**	**Low**			**High**			**Low vs. High**	
	**EC50**	**pEC50 ± s.e.m.**	**Emax ± s.e.m.**	**EC50**	**pEC50 ± s.e.m.**	**Emax ± s.e.m.**	**EC50**	**Emax**
**ISO**	6.17e-008	-7.21 ± 0.15	-0.058 ± 0.004	1.26e-007	-6.9 ± 0.13	-0.060 ± 0.003	ns	ns
**NE**	2.93e-007	-6.53 ± 0.14	-0.074 ± 0.005	2.49e-007	-6.60 ± 0.11	-0.081 ± 0.004	ns	ns
**EPI**	2.55e-006	-5.59 ± 0.17	-0.079 ± 0.01	1.28e-006	-5.89 ± 0.13	-0.072 ± 0.006	ns	ns

Supplementary Table 3

β2-AR

Gαs	Low			High			Low vs. High	
	EC50	pEC50 ± s.e.m.	Emax ± s.e.m.	EC50	pEC50 ± s.e.m.	Emax ± s.e.m.	EC50	Emax
ISO	2.12e-008	-7.67 ± 0.48	-0.017 ± 0.002	7.64e-010	-9.11 ± 0.3295	-0.023 ± 0.001	0.04*	ns
NE	8.03e-007	-6.09 ± 0.51	-0.018 ± 0.004	8.29e-008	-7.08 ± 0.3462	-0.022 ± 0.002	ns	ns
EPI	1.67e-009	-8.77 ± 0.41	-0.017 ± 0.002	8.96e-009	-8.05 ± 0.2458	-0.019 ± 0.001	ns	ns
Gαi1	Low			High			Low vs. High	
	EC50	pEC50 ± s.e.m.	Emax ± s.e.m.	EC50	pEC50 ± s.e.m.	Emax ± s.e.m.	EC50	Emax
ISO	1.61e-008	-7.79 ± 0.55	-0.057 ± 0.006	4.95e-008	-7.30 ± 0.27	-0.044 ± 0.005	ns	ns
NE	1.92e-007	-6.71 ± 0.27	-0.073 ± 0.008	6.49e-008	-7.19 ± 0.37	-0.064 ± 0.008	ns	ns
EPI	1.33e-007	-6.87 ± 0.42	-0.097 ± 0.01	5.83e-008	-7.23 ± 0.29	-0.063 ± 0.006	ns	0.04*
GαoA	Low			High			Low vs. High	
	EC50	pEC50 ± s.e.m.	Emax ± s.e.m.	EC50	pEC50 ± s.e.m.	Emax ± s.e.m.	EC50	Emax
ISO	3.48e-007	-6.46 ± 0.29	-0.041 ± 0.004	1.22e-007	-6.91 ± 0.25	-0.026 ± 0.002	ns	0.01*
NE	2.78e-007	-6.55 ± 0.29	-0.044 ± 0.005	5.34e-008	-7.27 ± 0.34	-0.028 ± 0.003	ns	0.03*
EPI	2.03e-007	-6.69 ± 0.21	-0.044 ± 0.003	5.07e-008	-7.29 ± 0.32	-0.030 ± 0.003	ns	0.04*

SUPPLEMENTARY FIGURE/TABLE LEGENDS

Supplementary Figure 1. β2-AR agonist maximal efficacy on endogenous β-receptor-mediated G protein activation. a-c) BRET in HEK293T cells expressing low (back bars) and high (orange bars) expression levels Gαs-Rluc8 (**a**), Gαi1-Rluc8 (**b**) or GαoA-Rluc8 (**c**) in presence of GFP10-Gγ2 and Gβ3 untagged subunits. Cells were stimulated or not for 1 min with 10 µM of the indicated agonists. Results are expressed as the difference in BRET signals measured in presence and absence of ligand. Data represent the mean ± s.e.m. of at least four independent experiments. The statistical significance between stimulated and unstimulated cells was assessed using paired Student's t-test (**P*<0.05, ***P*<0.01, ****P*<0.001). *Inset*: Relative expression levels of Gα-Rluc8 proteins reflected by the Rluc8 luminescence measured in each experimental transfections.

Supplementary Figure 2. Expression of the Gαs-Rluc8 over-expressed protein. Gαs expression was analyzed by western-blot from HEK293T cells expressing untagged-Gαs or different levels of Gαs-Rluc8 along with Gβ3 and GFP10-Gγ2 in the presence or not of the β1-AR or β2-AR or the empty pcDNA3.1 vector (control) as indicated. GAPDH was analyzed as housekeeping protein. The blot is representative of two independent experiments.

Supplementary Figure 3. Ligand efficacies parallels Gαi1 protein activation and conformational changes within β-AR/Gαi1 complexes. BRET measuring **a**) G protein activation in HEK293T cells expressing low level of Gαi1-Rluc8 in the presence of GFP10-Gγ2 and Gβ3 untagged subunit in the presence of β1-AR (gray bars) or β2-AR (black bars). or, **b**) conformational changes of β-AR/Gαi1 complexes in HEK293T cells co-expressing β1-AR-GFP10 (gray bars) or β2-AR-GFP10 (black bars) receptors in the presence of low level of Gαi1-Rluc8 along with untagged Gγ2 and Gβ3 subunits. Cells were stimulated or not for 1 min with 10 µM of the indicated agonists. Results are expressed as the difference in BRET signals measured in presence and absence of ligand. Data represent the mean ± s.e.m. of at least four independent experiments. The statistical significance between stimulated and unstimulated cells was assessed using paired Student's t-test (* *P*<0.05, ** *P*<0.01, *** *P*<0.001). **c, d**) Graphical illustration of the correlation between Gαi1 protein activation and β2-AR/Gαi1 conformational changes in cells expressing (**c**) or not (**d**) the β1-AR or β2-AR receptors.

Supplementary Figure 4. Concentration-response curves of cAMP production mediated by β-AR agonists. a, b) Quantification of cAMP levels performed in HEK293T cells co-expressing or not β1-AR (**a**) or β2-AR (**b**) receptors in the presence or not of low (black circles) or high (orange circles) expression levels of Gαs-Rluc8 along with GFP10-Gγ2 and Gβ3 subunits as in Fig. 3 and stimulated or not with various NE (left panels) or EPI (right panels) concentrations. The statistical significance between dose-response curves was assessed using two-way ANOVA followed by a Bonferroni posttest (**P*<0.05, ***P*<0.01). *Inset*: Relative expression levels of Gα-Rluc8 proteins reflected by the Rluc8 luminescence measured in each experimental transfection.

Supplementary Figure 5. Quantification of HA-β-AR cell surface expression by ELISA. **a, b**) ELISA experiments were performed on non-permeabilized HEK293T cells co-expressing HA-β1-AR (**a**) or HA-β2-AR (**b**) receptors and low (black) or high (orange) expression levels of Gαs-Rluc8, Gαi1-Rluc8 or GαoA-Rluc8 in presence of GFP10-Gγ2 and Gβ3 untagged subunit. Cell surface expression of β-ARs was quantified by ELISA using an anti-HA antibody. *Insets*: Each experimental transfection was calibrated and controlled for Gα-Rluc8 relative expression levels by measuring the total emission level of Rluc8 luminescence. Data represent the mean ± s.e.m. of three independent experiments. The statistical significance of the difference in cell surface receptor density between low and high Gα-Rluc8 expression level was assessed using unpaired Student's t-test.

Supplementary Figure 6. Relative expression level of Gα subunits in dose-response experiments. a-b) Relative expression levels of Gα-Rluc8 subunits reflected by the Rluc8 luminescence measured at basal state from **a, b**) HEK293T cells co-expressing HA-β1-AR (**a**) or HA-β2-AR (**b**) in the presence of low (black bars) or high (orange bars) Gαs-Rluc8, Gαi1-Rluc8 or GαoA-Rluc8 along with GFP10-Gγ2 and Gβ3 untagged subunit as shown in Fig. 3

Supplementary Figure 7. Influence of the Gα subunit expression level on the concentration-response curves of β-AR blockers-mediated G protein activation. **a-c**) BRET in HEK293T cells co-expressing HA-β1-AR (upper panels) or HA-β2-AR (lower panels) receptors and low (black) or high (orange) expression levels of Gαs-Rluc8 (**a**), Gαi1-Rluc8 (**b**) or GαoA-Rluc8 (**c**) in presence of fixed GFP10-Gγ2 and Gβ3 untagged subunits.

Cells were stimulated or not for 1 min with increasing concentrations of the indicated b-antagonists (Bisoprolol, BISO; Metoprolol, METO; Timolol, TIMO). Results are expressed as the difference in BRET signals measured in presence and absence of ligand. Data represent the mean ± s.e.m. of at least four independent experiments. The statistical significance between unstimulated cells and cells stimulated with the different ligand concentrations in low (*) and high ($) Gα-Rluc8 conditions was assessed using one-way ANOVA followed by a Dunnett's multiple comparison test (* $P<0.05$, ** $P<0.01$, *** $P<0.001$). The statistical significance between low and high dose-response curves was assessed using two-way ANOVA followed by a Bonferroni post-test (#$P<0.05$, ##$P<0.01$).

Supplementary Figure 8. Relative expression level of Gαi1 subunit in raft sucrose gradients. Relative expression levels of Gα-Rluc8 subunits reflected by the Rluc8 luminescence measured in each sucrose fractions from HEK293T cells co-expressing HA-β1-AR in the presence of two different high concentration Gαi1-Rluc8 along with GFP10-Gγ2 and Gβ3 untagged subunit.

Supplementary Figure 9. Membrane compartmentalization of the GABAB-R2 receptor. Sucrose gradient fractionation on HEK293T cells expressing the GABAB-R2-Rluc receptor. Results are expressed as the percentage of the total luminescence measured in each gradient. Grey box highlights raft nano-domains enriched fractions. Sucrose density of each fractions was quantified using a refractometer.

Supplementary Table 1. Potencies (EC50) and maximal efficacies (Emax) of Norepinephrine (NE) and Epinephrine (EPI) on cAMP production in HEK293T cells. Data were obtained from experiments shown in Supplementary Fig. 4 and represent the mean of EC50, pEC50 ± s.e.m. and Emax ± s.e.m..

Supplementary Table 2. Potencies (EC50) and maximal efficacies (Emax) of β-AR agonists on β1-AR-mediated Gαsβ3γ2, Gαi1β3γ2 and GαoAβ3γ2 activation in HEK293T cells. Data were obtained from experiments shown in Fig. 3 and represent the mean of EC50, pEC50 ± s.e.m. and Emax ± s.e.m. The statistical significance between low and high EC50 and Emax was assessed using unpaired Student's t-test (*$P<0.05$, **$P<0.01$, ns: not significant, na: not available).

Supplementary Table 3. Potencies (EC50) and maximal efficacies (Emax) of β-AR ligands on β2-AR-mediated Gαsβ3γ2, Gαi1β3γ2 and GαoAβ3γ2activation in HEK293T cells. Results were obtained from experiments shown in Fig. 3 and represent the mean of EC50, pEC50 ± s.e.m. and Emax ± s.e.m. The statistical significance between low and high EC50 and Emax was assessed using unpaired Student's t-test (**P*<0.05, ns: not significant).
